# Clinical perspectives on wearable devices for pediatric cyanotic congenital heart disease: an expert survey to inform the early development of a multiparametric wearable biosensor

**DOI:** 10.3389/fmed.2026.1819360

**Published:** 2026-05-19

**Authors:** Roberta Nossa, Antonio Lopalco, Angela Assunta Lopedota, Małgorzata Syczewska, Sandra Brasil, Eleonora Sarracco, Emanuela Crea, Francesco La Penna, Rosa Conte, Marina Montanaro, Giovanni Migliaccio, Marek Migdal, Franco Bartoloni, Donato Bonifazi, Emilia Biffi

**Affiliations:** 1 Scientific Institute, IRCCS Eugenio Medea, Bosisio Parini, Italy; 2 Department of Pharmacy – Pharmaceutical Sciences, University of Bari Aldo Moro, Bari, Italy; 3 The Children’s Memorial Health Institute (CMHI), Warsaw, Poland; 4 EPTRI - European Paediatric Transnational Research Infrastructure, University of Leuven, Leuven, Belgium; 5 TEDDY European Network of Excellence for Paediatric Clinical Research, Bari, Italy; 6 Fondazione per la Ricerca Farmacologica Gianni Benzi Onlus, Valenzano, Italy; 7 CVBF - Consorzio per Valutazioni Biologiche e Farmacologiche, Bari, Italy

**Keywords:** clinical expert survey, cyanotic congenital heart disease, multiparametric biosensor, orphan medical devices, pediatric cardiology, pediatric medical device development, remote patient monitoring, wearable devices

## Abstract

**Introduction:**

Pediatric medical device development remains limited, particularly for rare and complex conditions such as cyanotic congenital heart disease (CCHD). Although advances in surgery and diagnostics have improved survival, continuous and non-invasive monitoring of metabolic and physiological deterioration is not available outside hospital settings. Wearable devices could address this gap, but their clinical adoption requires alignment with real-world needs, usability, and healthcare workflows. This study aimed to capture clinician perspectives to inform the early development of a wearable multiparametric biosensor for pediatric CCHD within the European OrphaDev4Kids project (EU4H-2023-PJ).

**Methods:**

A structured, web-based anonymous survey was distributed through EPTRI channels to reach pediatric cardiology experts across Europe, including members of the OrphaDev4Kids Clinical Expert Committee. The questionnaire addressed current monitoring practices in CCHD, unmet clinical needs, usability and compliance, data integration, training requirements, and data governance. Quantitative responses were analyzed using descriptive statistics, and qualitative feedback was descriptively reviewed.

**Results:**

Clinicians reported that current CCHD monitoring relies mainly on imaging and basic physiological parameters, with minimal assessment of metabolic markers and no availability of non-invasive metabolic monitoring. Infancy, both before and after heart surgery, was identified as the most critical phase for remote monitoring. Respondents strongly supported non-invasive, multiparametric wearable devices providing threshold-based alerts and longitudinal summaries rather than continuous data streams. Comfort, intuitive design, and minimal disruption to daily activities were key determinants of patient compliance, while integration with clinical workflows, training, and technical support were essential for adoption. Clinicians also emphasized the importance of secure, regulation-compliant data handling. Despite limited familiarity with advanced wearable devices, clear and consistent expectations regarding functionality and usability emerged.

**Conclusion:**

This expert consultation identifies a clear unmet need and strong clinical support for the development of wearable, multiparametric biosensors tailored to pediatric patients with CCHD. The findings provide actionable guidance for device design and highlight the importance of combining technological innovation with human-centered design, workflow integration, and robust data governance to enable future clinical adoption.

## 1 Introduction

The development of medical devices for pediatric populations remains a major unmet challenge in clinical innovation (1). Children present distinct physiological characteristics compared to adults, including differences in body size, growth trajectories, metabolic rates, and disease expression. These differences require tailored design, validation, and regulatory strategies (2). Despite these specific needs, most medical devices currently available are developed primarily for adult populations, with pediatric applications frequently relying on off-label adaptations. As a result, there is a limited availability of devices specifically designed, validated, and approved for children, particularly in the context of rare and complex diseases (3).

Congenital heart disease (CHD) affects approximately 8–9 per 1,000 live births, with cyanotic congenital heart disease (CCHD) accounting for roughly one quarter of all cases (4). Considering current birth rates in the European Union, the number of new CCHD patients falls below the threshold defined for orphan populations by the Medical Device Coordination Group (MDCG 2024-10) (5). From a medical device perspective, an orphan population is defined as a patient population affected by a condition that occurs in not more than 12,000 individuals per year in the European Union, for which either there is an insufficient availability of alternative options or the available device is expected to provide a clinical benefit compared to the state of the art, taking into account device- and population-specific factors (5). CCHD encompasses a group of severe congenital cardiac malformations that impair systemic oxygenation through intracardiac shunts or outflow tract obstructions, leading to the circulation of deoxygenated blood. These conditions typically manifest early in life with persistent or episodic cyanosis and require complex, lifelong management (6).

Traditional monitoring of clinical status and physiology in patients with CCHD relies on a combination of laboratory tests (e.g., blood gas analysis, lactate, and electrolytes), imaging studies such as echocardiography and computed tomography, and routine vital sign assessments (e.g., heart rate and oxygen saturation) (7, 8). These parameters are clinically relevant as they reflect early alterations in oxygen delivery, hemodynamic instability, and systemic hypoperfusion, which are key drivers of deterioration in pediatric CCHD. In particular, increases in lactate and declines in oxygen saturation may precede overt clinical collapse and cardiovascular decompensation (8). However, CCHD physiology is often highly dynamic, especially during early life and transitional phases of care, with rapid and sometimes unpredictable shifts in hemodynamic balance. Adverse events may occur outside the hospital setting and are not always preceded by easily detectable clinical signs (7). Current monitoring strategies combine scheduled clinical evaluations with limited home-based measurements (e.g., pulse oximetry or weight tracking), providing only partial insight into the patient’s condition and potentially missing early signs of deterioration. More broadly, clinical decision-making continues to rely on periodic imaging, functional assessments, and symptom reporting, which may not fully capture transient or evolving physiological changes (7). Wearable biosensors offer a promising opportunity to overcome these limitations by enabling continuous, real-world monitoring of physiological parameters. This approach may allow the identification of patient-specific baselines and the early detection of clinically relevant deviations, supporting more timely intervention and improved management of pediatric patients with CCHD (7).

To date, no wearable devices specifically designed, clinically validated, and approved for real-time metabolic monitoring in pediatric patients with CCHD are available, reinforcing the classification of this technology as an orphan medical device (7) i.e., a device intended for the diagnosis, prevention, or treatment of a condition meeting the orphan population criteria as defined above under MDCG 2024-10 (5).

The OrphaDev4Kids European project (EU4H-2023-PJ) aims to address critical challenges in the development of orphan medical devices for pediatric populations by providing access to a comprehensive ecosystem of tools, facilities, and supportive services that accelerate innovation and development in this field. Within this framework, the project supports the development of three distinct device concepts, each targeting a different unmet clinical need. One of these concepts focuses on CCHD and proposes the development of a wearable multiparametric biosensor (i.e., a wearable device that simultaneously or sequentially measures multiple physiological parameters for integrated monitoring of health status) capable of real-time monitoring of metabolic and oxygenation parameters in both inpatient and outpatient pediatric populations.

As an initial step in this development pathway, the project sought expert clinical input to guide device design, define its intended role within existing care pathways, and inform implementation strategies, leading to the development of a structured survey addressed to pediatric cardiology professionals. This approach is consistent with the framework proposed by Tandon et al. (9), who provide a Science Advisory within the American Heart Association, synthesizing existing evidence and expert opinion on the opportunities, limitations, and implementation challenges of wearable biosensors in congenital heart disease, rather than generating primary data. In contrast, this article reports the methodology and findings of a structured expert consultation based on an anonymous web-based survey designed, distributed, and analyzed to capture clinicians’ perspectives on clinical utility, usability, patient compliance, and integration into existing healthcare workflows. The results provide early evidence to support informed design decisions and guide the future development stages of a novel wearable multiparametric biosensor for the management of pediatric patients with CCHD.

## 2 Materials and methods

### 2.1 Study design

This study adopted a structured expert-consultation design aimed at evaluating the clinical relevance, feasibility, and potential impact of a novel wearable multiparametric biosensor for pediatric patients with CCHD. The consultation was conducted as part of the early-stage development activities of the OrphaDev4Kids project and was intended to inform device design, define its intended clinical application and role within the care pathway, and guide subsequent development phases. The primary envisioned use scenario is home-based monitoring, with the aim of enabling early detection of clinical deterioration and supporting continuity of care outside the hospital setting; however, its use in inpatient and clinical environments is not precluded. To systematically capture expert input, a web-based survey was implemented and made available online, allowing respondents to submit responses between February 2025 and July 2025. A multi-step methodology was implemented to ensure the clinical robustness, relevance, and methodological transparency of the collected data.

### 2.2 Survey development

A comprehensive, structured questionnaire was developed to systematically capture expert opinions on key aspects of the proposed wearable multiparametric biosensor. The survey design was guided by three overarching objectives: (i) to assess the perceived clinical utility of real-time metabolic monitoring in pediatric CCHD; (ii) to evaluate usability, acceptability, and feasibility in real-world clinical and home settings; and (iii) to identify critical requirements for integration into existing healthcare workflows and information systems. Survey items addressed multiple dimensions of device development and implementation, including clinical monitoring needs, technological expectations, patient compliance, data management, and training requirements. A combination of closed-ended (Likert-scale, multiple choice) and open-ended questions was used to generate both quantitative and qualitative insights.

An initial version of the survey was drafted internally within the OrphaDev4Kids Consortium, which includes academic, clinical, and technological partners involved in the development of innovative solutions for pediatric rare diseases across Europe (Italy, Austria, Poland, and Belgium). It was subsequently circulated to core members of the project Clinical Expert Committee (CEC), a panel of clinicians, including pediatric cardiologists, cardiac surgeons, intensivists with recognized expertise in pediatric cardiology, involved in diagnosis, treatment, and long-term follow-up of patients with CCHD. Participation was voluntary, and experts were informed of the survey objectives and procedures. Feedback from the CEC focused on the clarity and clinical relevance of questions, the appropriateness of response options, and the logical organization of sections. Iterative revisions based on this input resulted in a finalized survey optimized to collect actionable and meaningful feedback from experts with diverse clinical backgrounds.

The final version of the questionnaire was implemented using REDCap (Research Electronic Data Capture), a secure, web-based platform widely adopted in clinical and academic research. REDCap was chosen for its robust security standards, flexible survey design, and comprehensive data management capabilities (10, 11). The system also facilitated real-time tracking of survey completion and secure data handling. Survey completion required approximately 20 min.

The survey was distributed through multiple channels, both within and beyond the OrphaDev4Kids Consortium, to maximize reach and participation. It was shared via LinkedIn, BlueSky and X (former Twitter) by the OrphaDev4Kids dissemination team and directly sent to CEC members, who were invited to participate as respondents. CEC members were also encouraged to forward the survey to colleagues with relevant expertise in pediatric cardiology, thereby expanding the pool of expert input and ensuring a broader perspective on clinical needs and wearable device requirements. The survey was also disseminated using MailChimp to different stakeholders including researchers and clinicians. The European Reference Network (ERN) GUARD-Heart was also contacted to help disseminate the survey among their members.

The study was conducted in compliance with applicable ethical and regulatory frameworks. No personal identifiers or sensitive data were collected, and all responses were recorded anonymously. Participants were informed that participation was voluntary and that they could discontinue at any time. In accordance with the General Data Protection Regulation (GDPR), the anonymous nature of data collection placed the study outside the scope of personal data processing requirements.

The full survey text is provided in the Supplementary Material.

#### 2.2.1 Survey structure and content

The questionnaire was organized into thematic sections designed to reflect the clinical decision-making process and operational context of CCHD management:

1. Expert profile and clinical context of CCHD management, capturing respondents’ role, years of experience, and degree of involvement in CCHD care.
2. Monitoring methods, including routinely monitored parameters, tools used, and limitations of existing approaches.
3. Feedback on wearable devices, exploring familiarity with wearable devices, expectations, and preferred functionalities.
4. Patient compliance and usability, addressing comfort, design, wearability, and factors influencing adherence in pediatric populations.
5. Integration with healthcare systems, evaluating expectations regarding data visualization, alert systems, frequency of data transmission, and interoperability with electronic health records.
6. Privacy and security, assessing clinicians’ concerns and requirements for safe implementation.
7. Training and support, identifying educational resources required for healthcare professionals, patients, and caregivers.
8. General feedback, allowing experts to provide additional comments and to express interest in participating in subsequent development phases.

### 2.3 Data analysis

Survey data were analyzed with Microsoft Excel using descriptive statistics to summarize response distributions for closed-ended items. Open-ended responses were limited and only available when respondents selected the “Other” option; these were reviewed descriptively to identify clinically relevant insights. Given the limited volume of qualitative input, no formal thematic analysis was performed. Results are presented as aggregated data, with illustrative expert comments used where appropriate to contextualize key findings.

## 3 Results

### 3.1 Expert profile and clinical context of CCHD management

A total of 19 clinicians agreed to participate in the survey, of whom 17 completed the questionnaire, resulting in a completion rate of 89.5%, while two participants did not complete the survey and were therefore classified as dropouts. Among the respondents who completed the survey, 64.7% were pediatric cardiologists, 23.5% general pediatricians, 5.9% cardiac surgeons, and 5.9% medical students, ensuring a multidisciplinary perspective on the management of CCHD.

**FIGURE 1:**
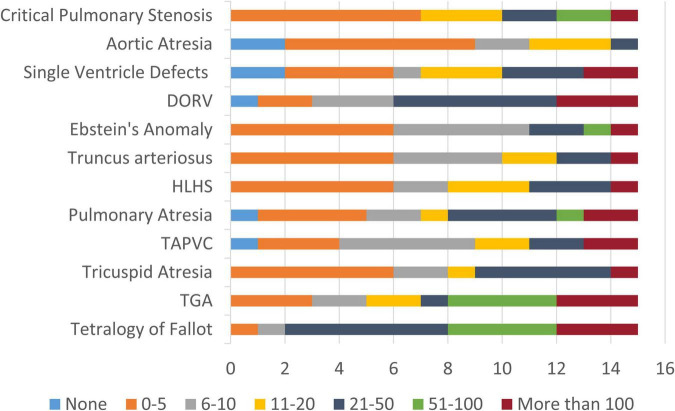
Number of patients reported by respondents with direct professional experience in managing each type of CCHD. The figure shows the distribution of patient volumes reported by clinicians (***n*** = 15) across different cyanotic congenital heart diseases. TGA, Transposition of the Great Arteries; TAPVC, Total Anomalous Pulmonary Venous Connection; HLHS, Hypoplastic Left Heart Syndrome; DORV, Double Outlet Right Ventricle.

Of the 17 respondents, 15 clinicians (88.2%) indicated direct professional experience in managing patients with CCHD, confirming the strong clinical relevance of the expert panel. Among them, more than half (53.3%) had over 10 years of experience working with these patients, while 33.3% had 6–10 years and 13.3% had 1–5 years of experience, indicating a high overall level of expertise within the group. These clinicians encounter patients with CCHD across a broad age range. The most frequently represented age group was 1–5 years (82.4%), followed by 0–1 year, 6–12 years, and 13–17 years (each indicated by 76.5% of respondents). Experience with adult patients (>18 years) was also noted by 35.3% of clinicians, reflecting continuity of care across the lifespan and reinforcing the relevance of long-term monitoring solutions. Regarding patient volume, most manage between 0 and 5 patients for the majority of CCHD conditions, followed by those caring for 21–50 and 6–10 patients (Figure 1). Notably, a subset demonstrated extensive clinical exposure, with experience ranging from 51–100 to more than 100 patients for specific conditions.

**FIGURE 2:**
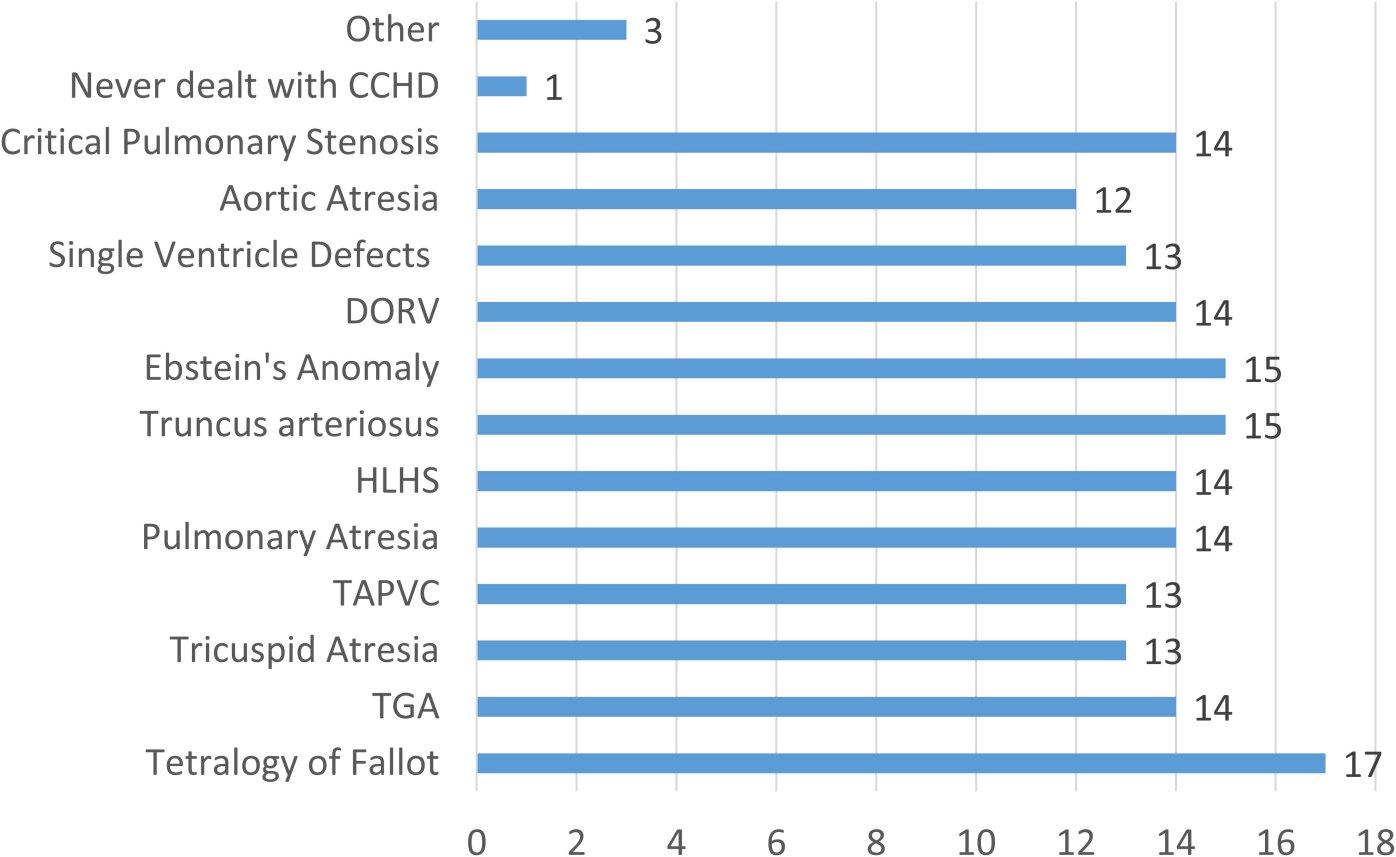
Distribution of CCHD conditions reported by survey respondents across their professional experience (clinical, research, and academic settings). Bars represent the number of clinicians reporting experience with each condition (*n* = 17). TGA, Transposition of the Great Arteries; TAPVC, Total Anomalous Pulmonary Venous Connection; HLHS, Hypoplastic Left Heart Syndrome; DORV, Double Outlet Right Ventricle.

Among the 17 respondents who indicated professional involvement CCHD, although not necessarily through direct patient management, familiarity with a broad range of CCHD conditions was reported through clinical practice, research activities, or academic investigations, as shown in Figure 2. All clinicians indicated professional experience with Tetralogy of Fallot. Experience with Transposition of the Great Arteries (TGA), Pulmonary Atresia, Hypoplastic Left Heart Syndrome (HLHS), Double Outlet Right Ventricle (DORV), and Critical Pulmonary Stenosis was reported by 82.4% of respondents. Seventy-six percent reported familiarity with Tricuspid Atresia, Total Anomalous Pulmonary Venous Connection (TAPVC), and single-ventricle defects, while 70.6% had experience with Aortic Atresia. Furthermore, 17.6% of respondents selected the “other” option, reporting experience with intra-atrial and interventricular defects or indicating exposure to the entire spectrum of congenital heart diseases, further underscoring the breadth of clinical expertise represented in the panel.

**FIGURE 3:**
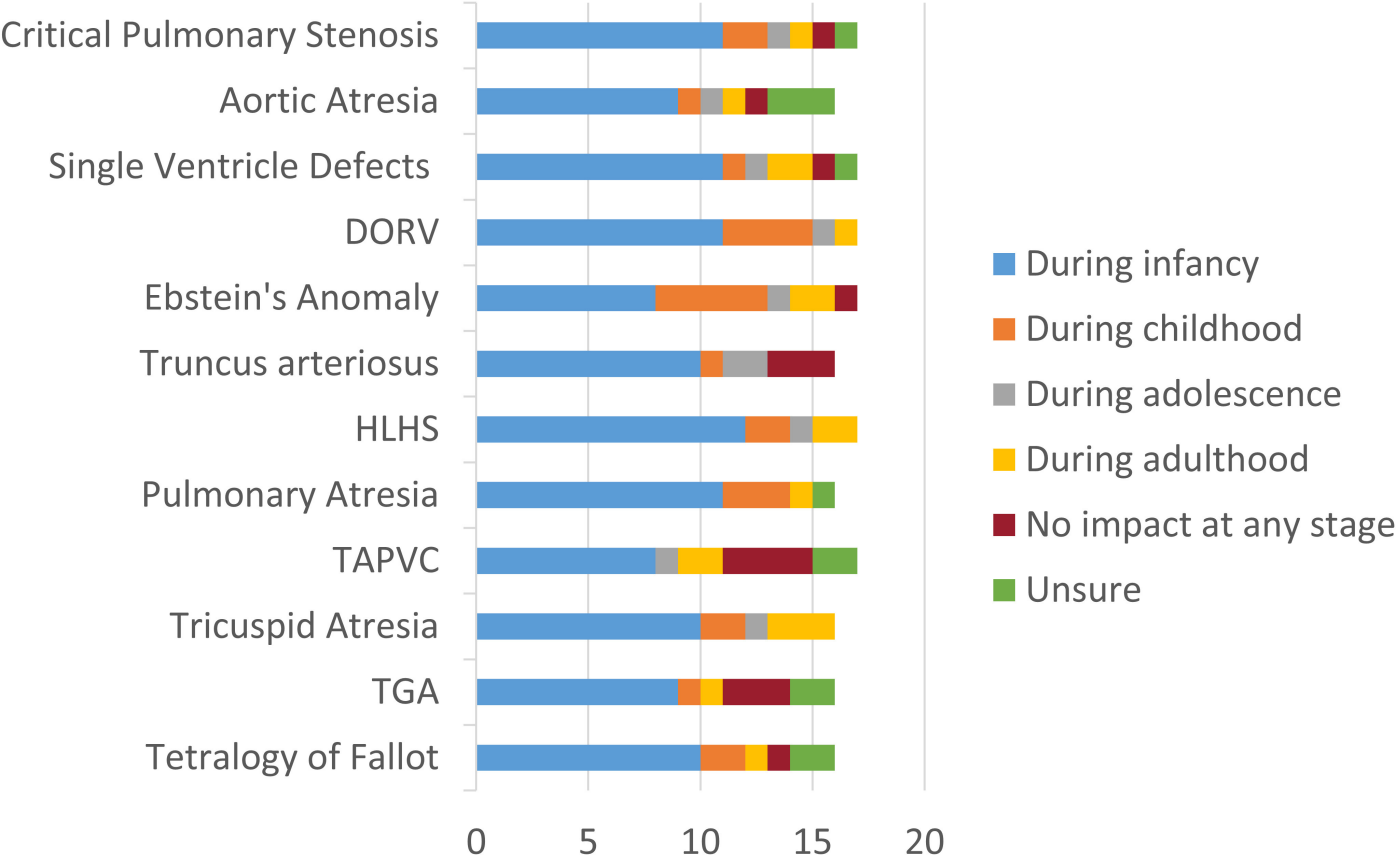
Most impactful stage for remote monitoring of patients with CCHD. The figure shows clinicians’ perceptions of the disease stage at which remote or non-invasive monitoring would provide the greatest clinical benefit across different CCHD conditions (*n* = 17). TGA, Transposition of the Great Arteries; TAPVC, Total Anomalous Pulmonary Venous Connection; HLHS, Hypoplastic Left Heart Syndrome; DORV, Double Outlet Right Ventricle.

When asked to identify the stage at which non-invasive or remote monitoring would provide the greatest clinical benefit, clinicians consistently indicated infancy as the most critical window across nearly all CCHD conditions, both before and after initial surgical repair (Figure 3). This finding underscores the potential life-saving role of early non-invasive metabolic and physiological monitoring during a particularly vulnerable phase of development. However, one respondent provided a more cautious perspective, noting that the impact on mortality might be limited, as high-risk patients are already closely monitored during clinical follow-up and acute events such as Fallot spells are rare when timely surgical repair is performed.

Beyond clinical management, experts identified major challenges affecting the daily lives of children with CCHD and their families. The most frequently reported issues were limitations in physical activity (76.5%), frequent medical visits (70.6%), and psychological stress (64.7%), followed by difficulties related to medication management (23.5%).

### 3.2 Monitoring methods

**FIGURE 4:**
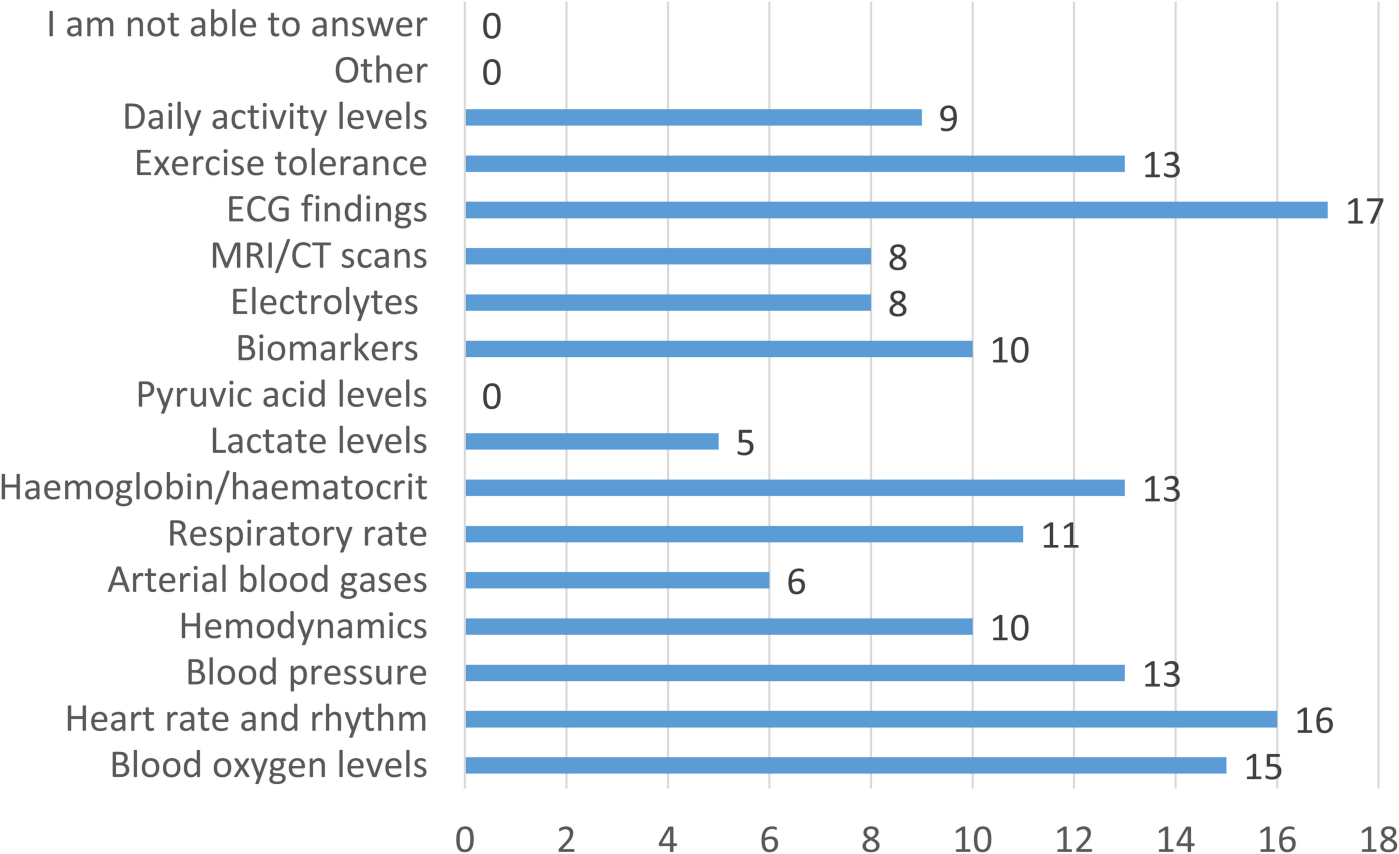
Frequency of physiological, biochemical, and imaging parameters monitored in patients with CCHD as part of standard clinical care. Bars represent the number of respondents reporting routine monitoring of each parameter (*n* = 17).

According to clinicians, standard clinical monitoring of patients with CCHD is predominantly focused on imaging assessments and basic physiological measures, as shown in Figure 4. Echocardiographic findings were selected by all respondents as a monitoring method, confirming their central role in routine care. This was followed by heart rate and rhythm (94.1%) and blood oxygen saturation (88.2%). Blood pressure, hemoglobin/hematocrit, and exercise tolerance were each selected by 76.5% of clinicians, while respiratory rate was selected by 64.7%. Hemodynamic parameters such as cardiac output were selected by 58.8% of respondents, followed by biomarkers (58.8%), daily activity levels (52.9%), electrolytes (47.1%), and MRI/CT imaging (47.1%). Arterial blood gases were selected by 35.3% of clinicians, lactate levels by 29.4%, and pyruvic acid was not selected by any respondent, highlighting a substantial gap in metabolic monitoring. Qualitative comments provided additional context on how some of these parameters are assessed in practice. Daily activity levels were mainly evaluated through indirect or subjective measures, including cardiopulmonary exercise testing (CPET), heart rate and oxygen saturation during activity, standardized questionnaires (e.g., International Physical Activity Questionnaire, Satisfaction and Quality of Life questionnaire), school and home activity reports, medical history, and patients’ or parents’ subjective perceptions of functional capacity (e.g., ability to climb stairs or participate in sports). Exercise tolerance, when assessed, was primarily evaluated using exercise testing, CPET, or functional classifications such as NYHA (New York Heart Association). Respondents also clarified that arterial blood gases are measured only rarely, typically in intensive care settings, due to the need for invasive sampling. Additional parameters occasionally monitored outside routine protocols included indicators of metabolic syndrome and selected biochemical markers such as CA-125 and protein C. Finally, one clinician commented that in most cyanotic CHDs, except univentricular hearts, early surgical repair in infancy reduces the need for complex monitoring beyond the first 6–12 months of life, reflecting differences in long-term follow-up strategies across diagnoses.

**FIGURE 5:**
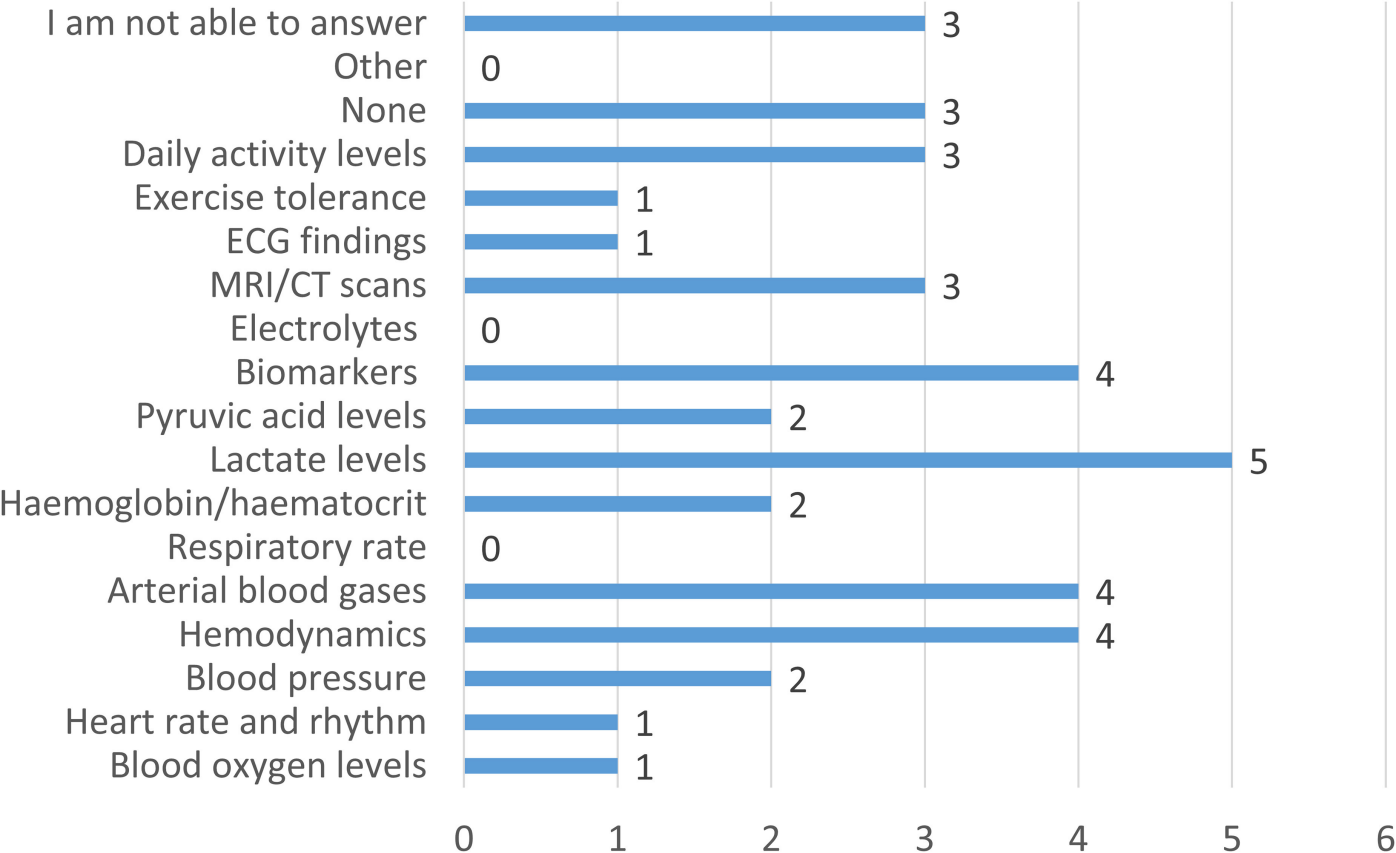
Additional physiological and biochemical parameters considered valuable for the management of patients with CCHD but not currently monitored or not widely adopted in standard care. Bars indicate the number of respondents selecting each parameter (*n* = 17).

When clinicians were asked which additional parameters, currently not monitored or not widely adopted, would be valuable for managing CCHD, responses showed considerable variability in the number of parameters selected. Nearly half of the respondents (47.4%) identified one additional parameter, while 21.0% selected three parameters. Smaller proportions indicated no additional parameters (10.5%) or four parameters (10.5%), whereas 5.3% selected two parameters and another 5.3% identified as many as eight parameters. Among the specific options reported in Figure 5, lactate levels were most frequently selected (29.4%). This was followed by hemodynamic parameters such as cardiac output (23.5%), arterial blood gases (23.5%), and circulating biomarkers (23.5%). Daily activity levels and MRI/CT imaging were each identified by 17.6% of respondents, while pyruvic acid and hemoglobin/hematocrit were selected by 11.8%. Only small proportions considered exercise tolerance, oxygen saturation, heart rate, echocardiography, or blood pressure as missing parameters (≤11.8%). Notably, none of the respondents selected respiratory rate or electrolytes (e.g., sodium, potassium) as additional parameters of interest.

**FIGURE 6:**
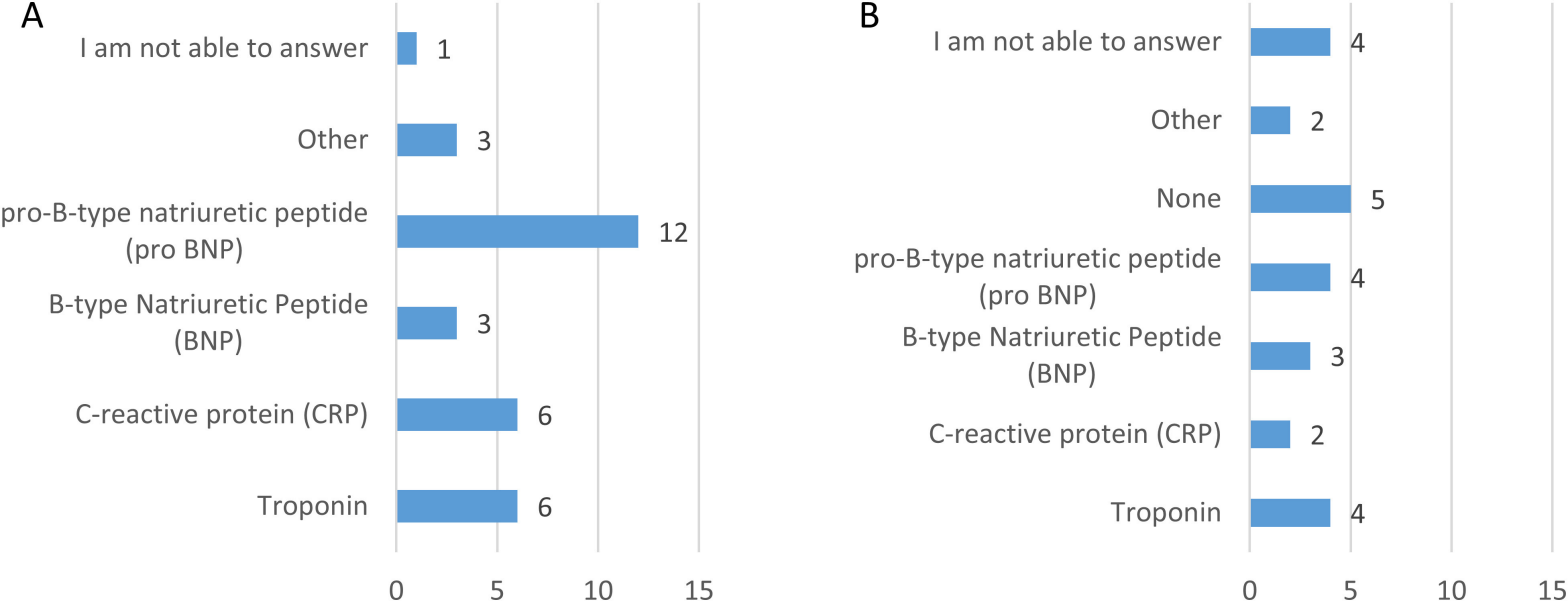
Biomarkers monitored in standard clinical practice and additional biomarkers considered potentially useful for patients with CCHD. Panel **(A)** shows the biomarkers currently monitored as part of standard care, while Panel **(B)** presents the biomarkers that respondents considered useful to monitor beyond those currently available. Bars represent the number of respondents selecting each biomarker (*n* = 17).

Regarding biomarker monitoring in standard care (Figure 6A), pro–B-type natriuretic peptide (pro-BNP) was the most frequently assessed biomarker (70.6%), followed by troponin (35.3%) and C-reactive protein (CRP, 35.3%). B-type Natriuretic Peptide (BNP) was monitored by 17.6% of clinicians, while 17.6% reported monitoring other biomarkers such as hemoglobin. When asked which biomarkers would be useful to monitor beyond those currently available (Figure 6B), pro-BNP and troponin were again most frequently selected (both 23.5%), followed by BNP (17.6%) and CRP (11.8%). In addition, 11.8% of respondents selected the option “other,” suggesting additional biomarkers such as CA-125, serum iron, and vitamin D levels. Notably, 29.4% of clinicians indicated that no additional biomarkers were needed, and 23.5% were unsure, reflecting some uncertainty regarding the clinical added value of expanded biomarker panels beyond those already used in routine practice.

Most clinicians reported that the biomarkers used in CCHD management cannot currently be assessed through methods other than blood sampling (47.1%), while 41.2% were uncertain. Only 11.8% indicated that non-blood-based alternatives are available, confirming the lack of non-invasive biochemical monitoring options in routine care.

**FIGURE 7:**
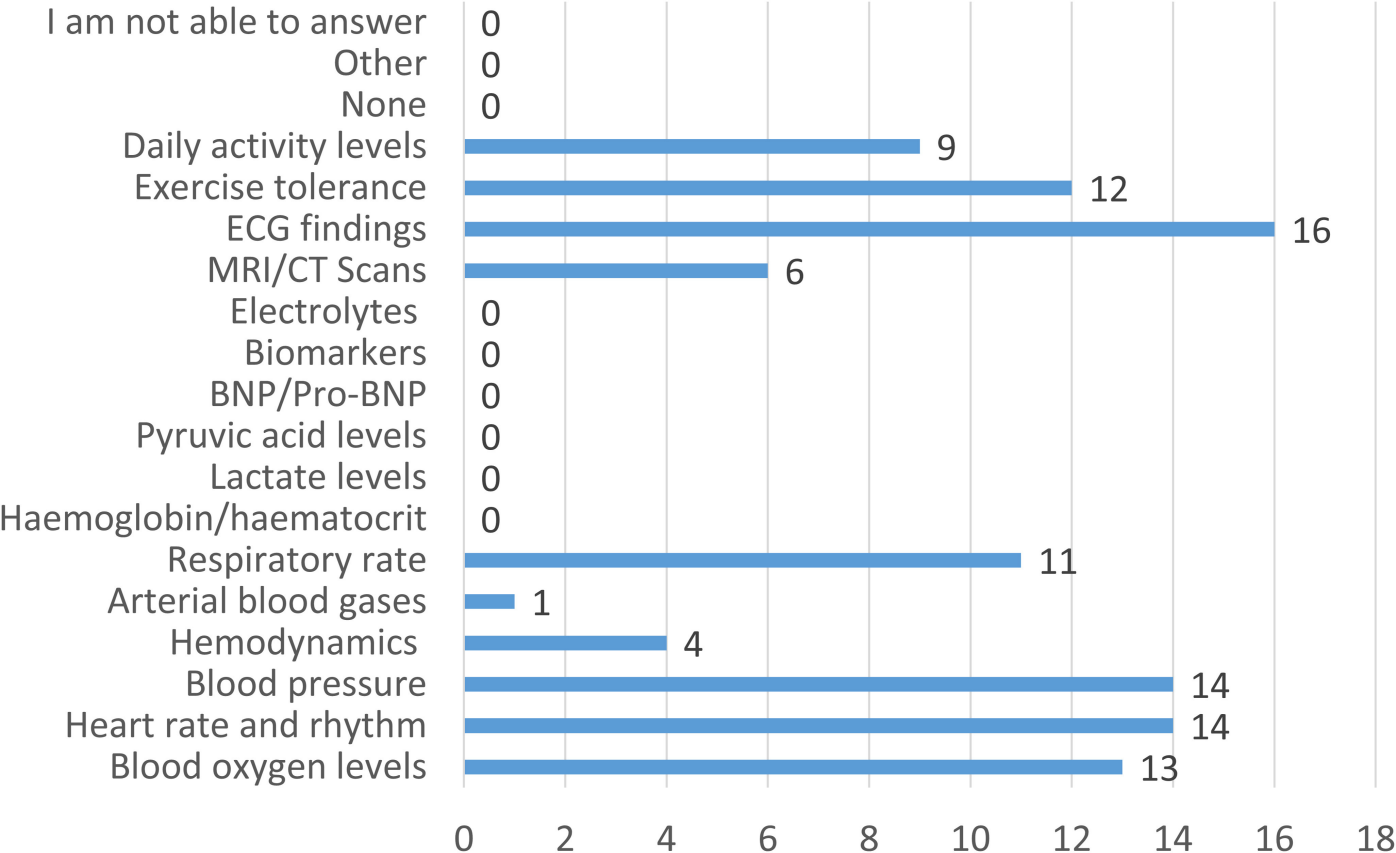
Physiological, functional, and biochemical parameters monitored using non-invasive methods in patients with CCHD. Bars represent the number of respondents reporting non-invasive assessments for each parameter (*n* = 17).

When asked which physiological and biochemical parameters currently monitored in patients with CCHD as part of standard care are assessed using non-invasive methods, respondents indicated that non-invasive monitoring primarily involves physiological and functional parameters (i.e., exercise tolerance and daily activity levels), as shown in Figure 7. Echocardiographic findings were the most frequently monitored non-invasively (94.1%), followed by heart rate and rhythm (82.4%), blood pressure (82.4%), and oxygen saturation (76.5%). Exercise tolerance (70.6%), respiratory rate (64.7%), and daily activity levels (52.9%) were also commonly assessed using non-invasive methods. Imaging techniques such as MRI/CT scans were reported as non-invasive monitoring tools by 35.3% of respondents, reflecting their role in structural and functional assessment rather than continuous monitoring. In contrast, non-invasive assessment of hemodynamic parameters was reported by only 23.5% of clinicians, while arterial blood gases (5.9%) and biochemical or metabolic markers–including lactate, pyruvate, hemoglobin/hematocrit, electrolytes, and cardiac biomarkers–were not monitored non-invasively by any respondent.

Current monitoring methods, both invasive and non-invasive, were perceived as very effective by 58.8% of clinicians and somewhat effective by 35.3%, with only 5.9% expressing a neutral opinion. When clinicians were asked to identify the main limitations of current monitoring approaches, the most frequently reported issues were the lack of integration with existing healthcare information systems (35.3%) and patient discomfort associated with device use (35.3%). These were followed by delayed feedback (17.6%), referring to the inability to obtain timely or actionable information to support prompt clinical decision-making. High costs and technical issues, such as connectivity problems, were reported less frequently (both 11.8%). Only a small proportion of respondents (5.9%) identified the limited range of parameters currently monitored as a major limitation. Notably, 17.6% of clinicians reported no relevant limitations with current monitoring strategies. Among the 17.6% who selected the option “other,” respondents highlighted that monitoring is often limited to specific time points rather than being continuous, resulting in a lack of repetitive or longitudinal information and reducing the ability to detect early clinical deterioration. Additionally, clinicians noted that the most sensitive early indicators of deterioration may differ across CCHD diagnoses, further emphasizing the limitations of intermittent and non-personalized monitoring approaches.

Opinions regarding the existence of non-invasive monitoring alternatives not yet widely adopted were mixed: 47.1% of clinicians were unsure, 29.4% believed such alternatives exist, and 23.5% believed they do not, highlighting both openness to innovation and uncertainty regarding technological maturity.

**FIGURE 8:**
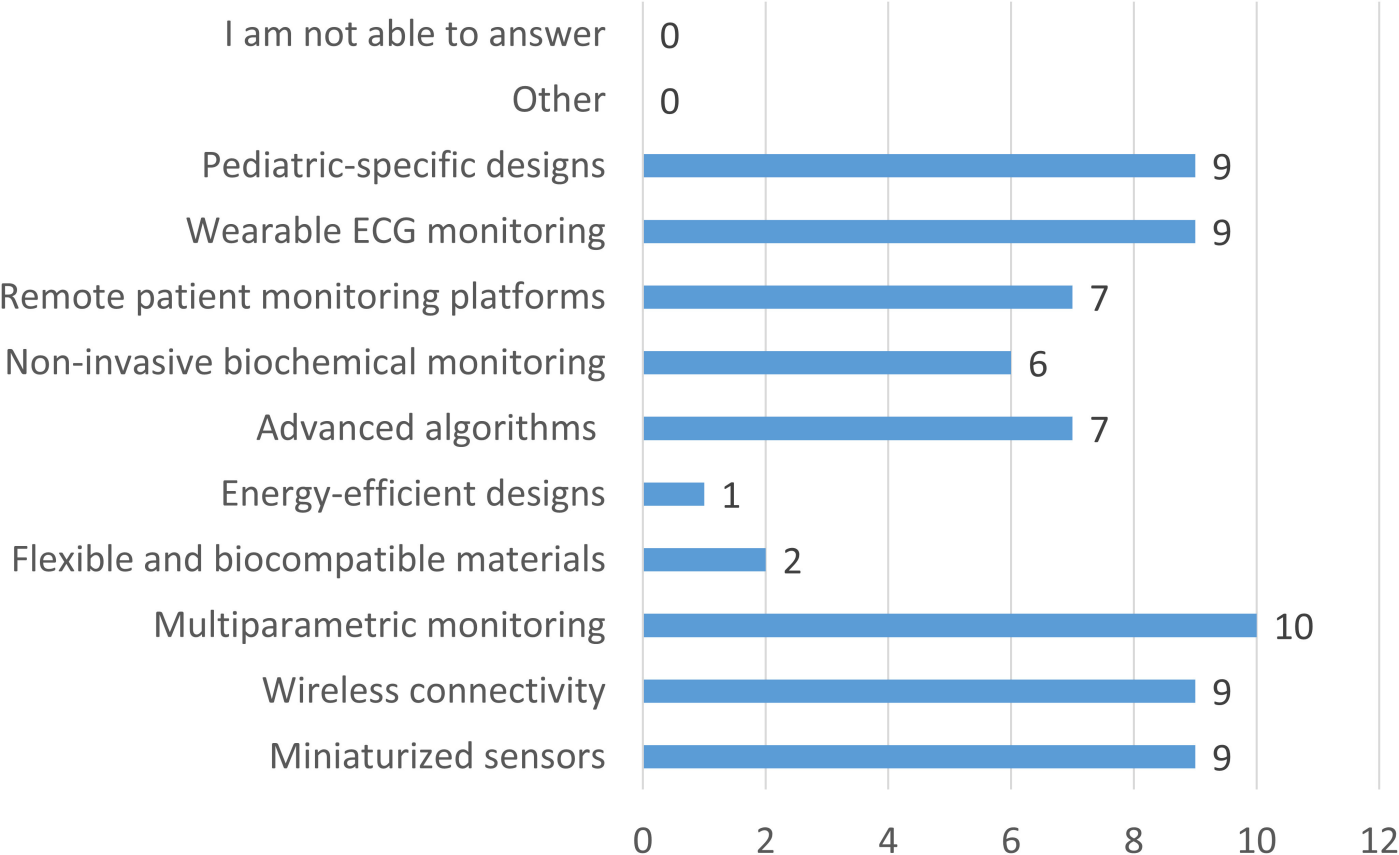
Prioritized advancements in wearable devices for monitoring patients with CCHD. Bars represent the number of clinicians selecting each technological feature as a priority for future development (*n* = 17).

When prioritizing future advancements in wearable devices for CCHD, clinicians most frequently selected multiparametric monitoring (58.8%) (Figure 8). Miniaturized sensors, wireless connectivity, wearable electrocardiogram (ECG) monitoring, and pediatric-specific designs were each selected by 52.9% of respondents. Advanced algorithms for early detection and personalized insights (41.2%), remote monitoring platforms (41.2%), and non-invasive biochemical monitoring (35.3%) were also highly prioritized, while flexible materials (11.8%) and energy-efficient designs (5.9%) were considered less critical.

**FIGURE 9:**
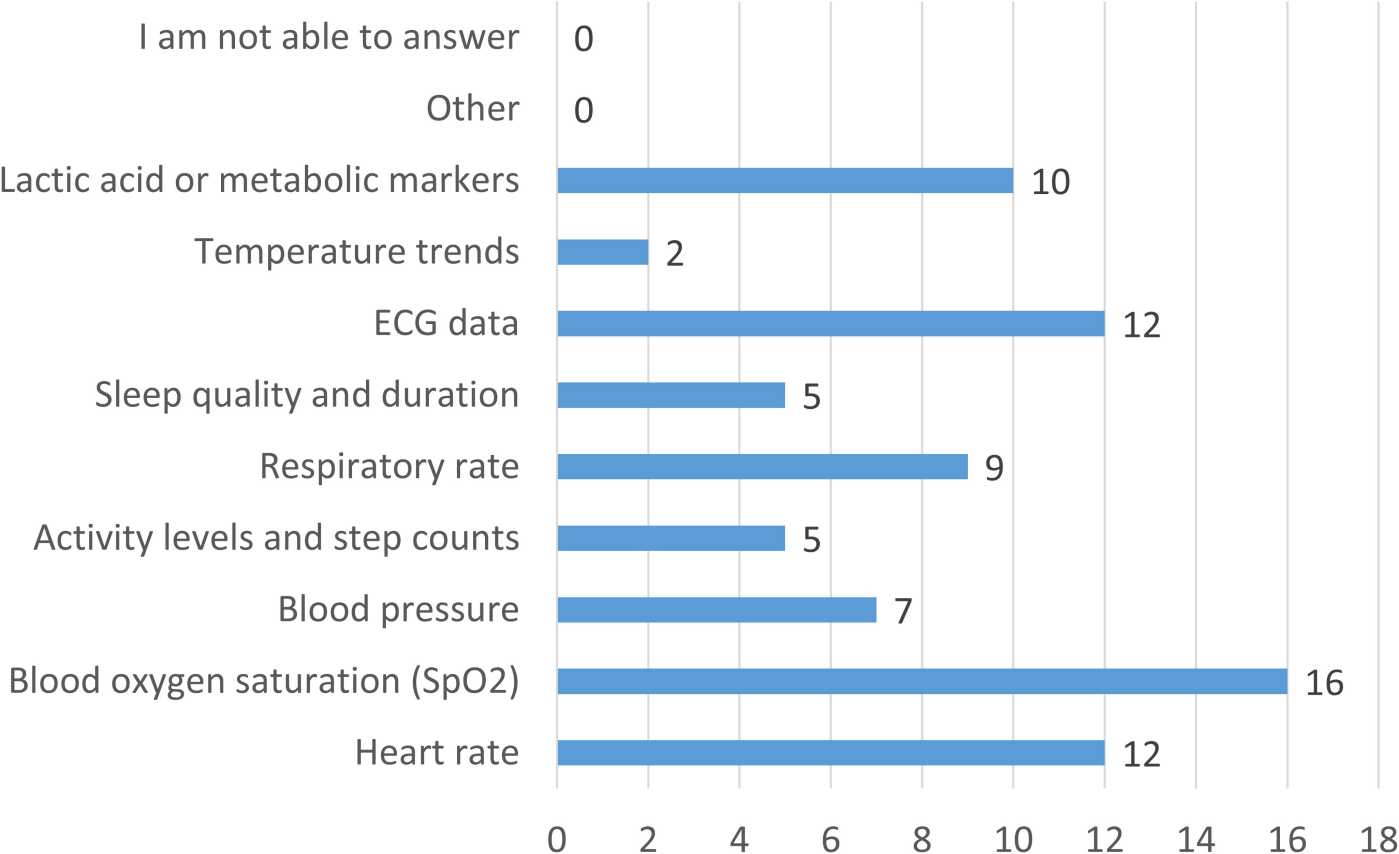
Types of physiological and biochemical data prioritized for collection by future wearable devices in CCHD care. Bars represent the number of clinicians indicating each parameter as a priority (*n* = 17).

In terms of data types to be prioritized by future wearable devices (Figure 9), blood oxygen saturation was selected by 94.1% of clinicians, followed by heart rate and ECG data (both 70.6%). Metabolic markers such as lactate (58.8%), respiratory rate (52.9%), and blood pressure (41.2%) were also frequently identified. Activity levels and sleep quality were each selected by 29.4%, while temperature trends were considered relevant by 11.8%.

Finally, clinicians expressed a clear preference for how wearable devices should deliver information: real-time updates were preferred by 35.3% of respondents, followed by threshold-based alerts (23.5%). Less frequent updates (hourly, daily, or weekly summaries) were each selected by smaller proportions (≤11.8%). Access to historical data and longitudinal trends was considered extremely important by 76.5% of clinicians and important by 17.6%, underscoring the critical role of continuous, time-resolved monitoring for clinical decision-making in CCHD.

### 3.3 Feedback on wearable devices

**FIGURE 10:**
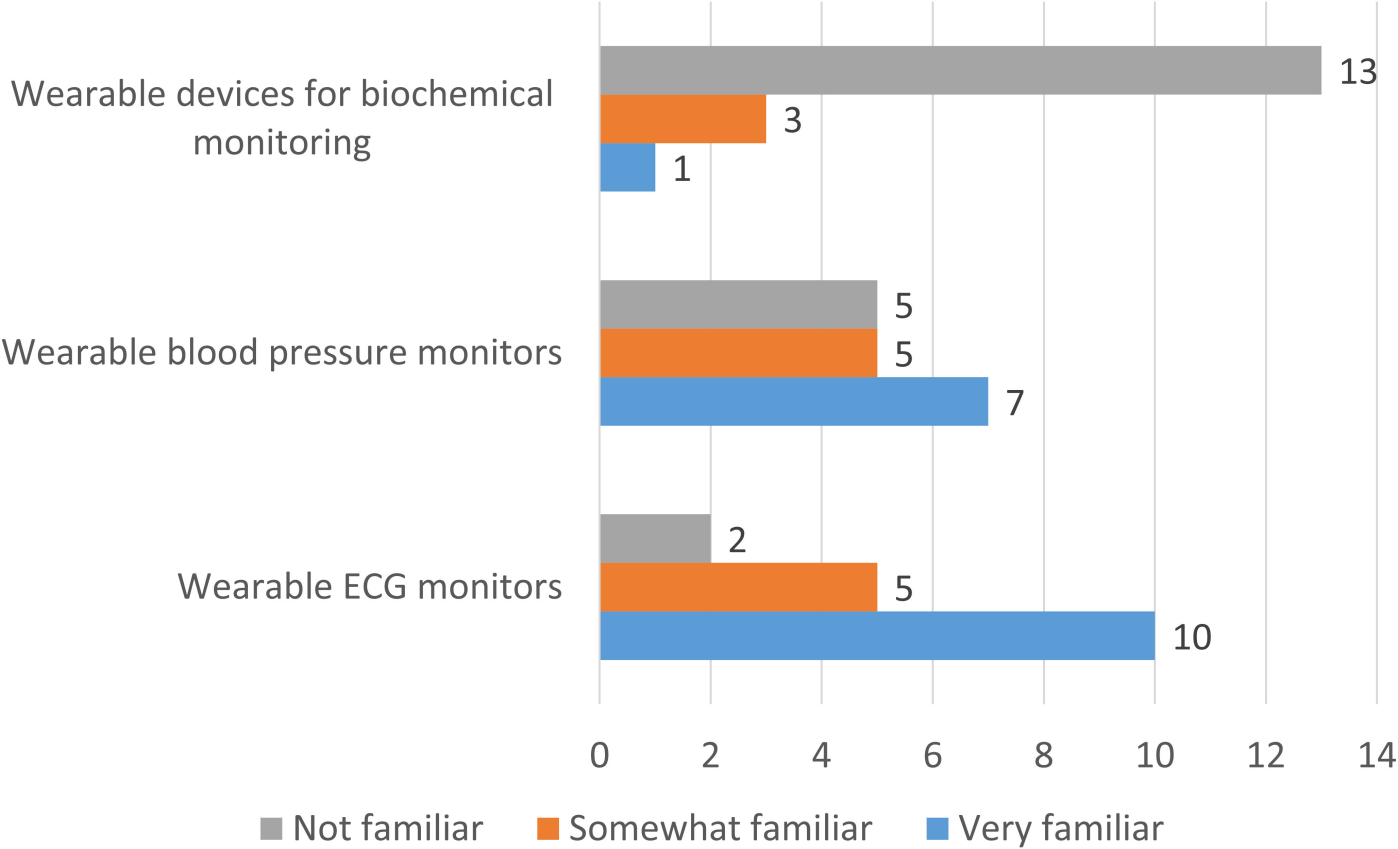
Clinicians’ familiarity with different types of wearable devices for pediatric cardiology. Bars represent the number of clinicians indicating familiarity with existing wearable devices (*n* = 17).

Clinicians reported heterogeneous levels of familiarity with existing wearable devices (Figure 10). Wearable ECG monitors were the most widely known, with 58.8% of respondents indicating that they were very familiar and 29.4% somewhat familiar, while only 11.8% reported no familiarity. Familiarity with wearable blood pressure monitors was more evenly distributed, with 41.2% very familiar, 29.4% somewhat familiar, and 29.4% not familiar. In contrast, wearable devices for biochemical monitoring (e.g., lactate, glucose, electrolytes) were largely unfamiliar: only 5.9% of clinicians reported being very familiar, 17.6% somewhat familiar, and the majority (76.5%) indicated no prior experience.

**FIGURE 11:**
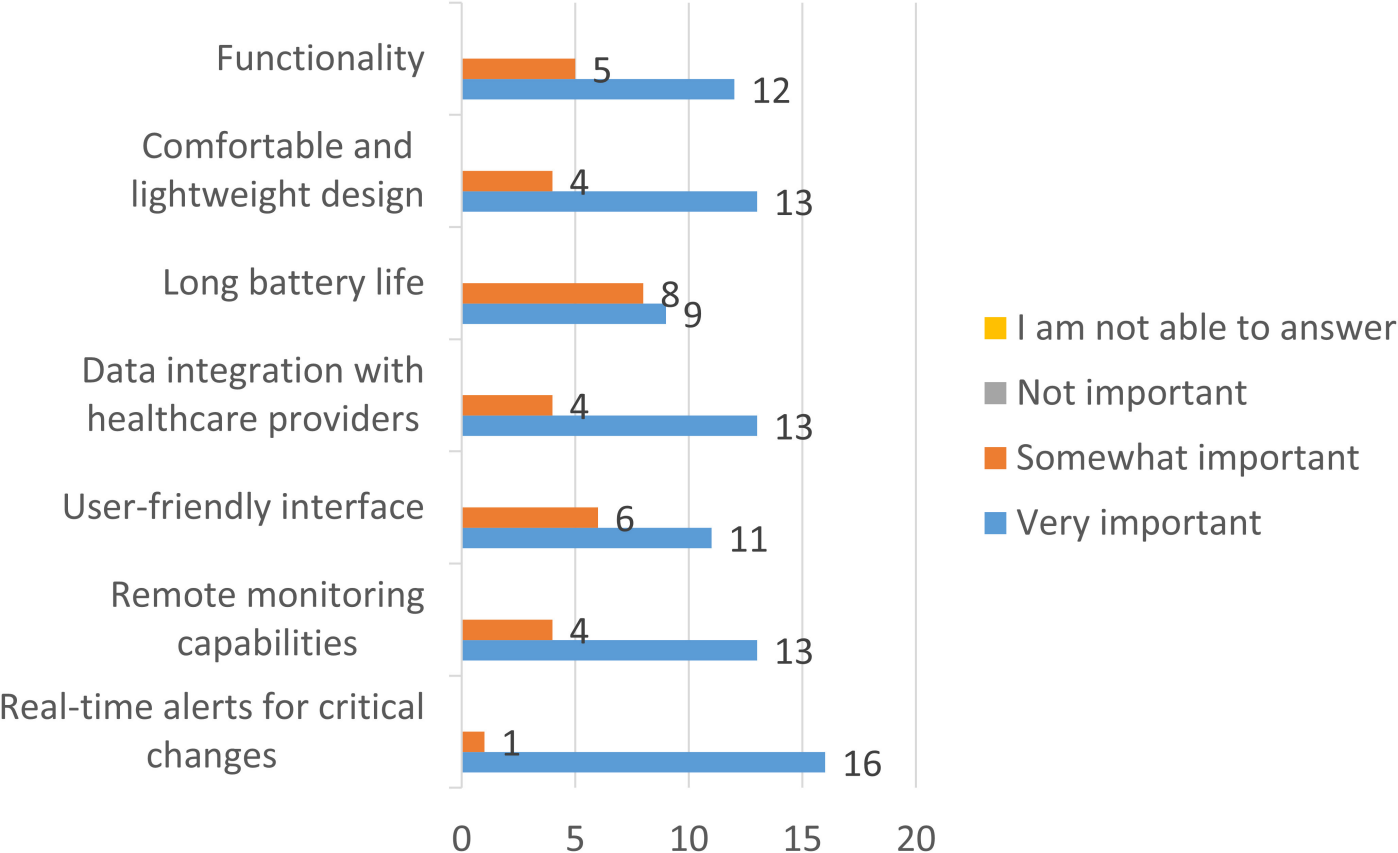
Importance of features for a wearable device designed for patients with CCHD. Bars represent the number of clinicians indicating consensus regarding the features they considered essential for a wearable device intended for CCHD monitoring (*n* = 17).

Despite limited familiarity with biochemical wearable devices, clinicians showed strong consensus regarding the features they considered essential for a wearable device intended for CCHD monitoring (Figure 11). Real-time alerts for critical physiological changes were rated as very important by 94.1% of respondents, followed by remote monitoring capabilities (76.5%), integration of data into healthcare providers’ systems (76.5%), and a comfortable, lightweight design suitable for pediatric use (76.5%). Functionality (70.6%) and user-friendly interfaces (64.7%) were also consistently prioritized. Long battery life, while still considered important, showed slightly more variability, with 52.9% of respondents rating it as very important and 47.1% as somewhat important, reflecting common trade-offs between device autonomy and form factor in wearable design.

### 3.4 Patient compliance and usability

Clinicians identified several factors influencing patient compliance with wearable devices. The most frequently selected determinant was comfort and design of the device (88.2%), followed by cost and associated maintenance (58.8%) and accuracy and reliability of data (41.2%). Additional factors included frequency of use (35.3%), awareness of the health benefits (35.3%), technical support and ease of troubleshooting (35.3%), psychological factors such as stigma or self-image concerns (35.3%), and parental involvement (29.4%). Comfort and ease of use emerged as particularly critical, with 82.4% of clinicians rating these aspects as extremely important and 17.6% as important for ensuring effective adoption in clinical settings.

When asked about the practical challenges patients may encounter in daily use, clinicians most frequently reported interference with daily activities (70.6%), followed by discomfort during prolonged wear (64.7%) and difficulty in operating or understanding the device (41.2%). Other notable barriers included psychological resistance (35.3%), limited battery life or need for frequent charging (35.3%), social stigma (23.5%), cost of replacement parts or consumables (17.6%), and lack of integration with other medical devices or platforms (11.8%) or concerns about data privacy and security (11.8%).

### 3.5 Integration with healthcare systems

The integration of patient-generated health data from wearable devices into routine clinical practice remains limited despite strong clinician interest. Indeed, most respondents (76.5%) reported that data are currently reviewed directly by healthcare providers during patient visits. Smaller proportions indicated that data are integrated into Electronic Health Records (EHR) for review (17.6%), reviewed by nursing or support staff before consultations (17.6%), used mainly for research or long-term trend analysis (5.9%), or not currently integrated due to technical or time constraints (11.8%).

All clinicians agreed that automatic sharing of data with healthcare systems would be useful. Regarding preferred frequency, the majority favored threshold-triggered alerts (47.1%) or daily summaries (29.4%), while both real-time continuous monitoring and hourly updates were each selected by 11.8% of respondents, reflecting concerns about potential data overload.

Clinicians prioritized the type of information they would like to receive from wearable devices to support clinical practice. Alerts for critical conditions, such as low oxygen levels, were considered most important (88.2%), followed by summaries of trends over specified periods (70.6%) and real-time patient feedback or notes on symptoms (47.0%). Integration of all available metrics into a single dashboard and data for specific activities, such as exercise, were each considered valuable by 35.3% of respondents.

Institutional readiness to handle data varied. Only 17.6% of respondents reported having a dedicated team for reviewing wearable device data, while 23.5% indicated that systems existed but were not fully integrated with clinical workflows. The majority (41.2%) cited insufficient capacity to manage real-time or frequent alerts, and 17.6% reported that their institution is considering developing such facilities.

Time availability for integrating wearable device data into routine care was limited. Most respondents (35.3%) indicated that clinical teams could dedicate 5–15 min per patient, while another 35.3% were uncertain and noted that it depends on the device and workflow integration. Smaller proportions reported less than 5 min (5.9%), 15–30 min (5.9%), or 30 min to 1 h (17.6%). No respondents expected to dedicate more than 1 h per patient.

### 3.6 Privacy and security

A substantial proportion of clinicians expressed high concern regarding the privacy and security of data collected by wearable devices. Specifically, 41.2% of respondents were very concerned, emphasizing the need for strict security protocols, while another 41.2% were somewhat concerned, provided that standard regulatory frameworks such as GDPR are fully respected. Only 17.6% expressed low concern, prioritizing patient benefit over potential privacy risks. Importantly, no respondent reported a lack of concern, underscoring that data protection is considered a critical requirement for the clinical adoption of wearable devices.

### 3.7 Training and support

Survey responses revealed a strong consensus among clinicians on the need for structured training and accessible support to enable effective adoption of wearable devices in clinical practice, with no respondent indicating that training would be unnecessary. Specifically, 64.7% of respondents selected comprehensive in-person training as an essential format. The same proportion (64.7%) also identified online video tutorials or e-learning modules as essential, and 64.7% indicated access to a technical support hotline or chat as equally important. In addition, 29.4% prioritized regular updates and refresher courses. This distribution highlights a preference for flexible, multimodal educational approaches that can accommodate diverse clinical roles and time constraints.

Regarding ongoing updates and technical support, 64.7% of clinicians preferred online resources such as webinars and manuals. In addition, 41.2% selected on-demand customer support, and the same proportion (41.2%) indicated automatic updates integrated into the device or software as important features. In-person workshops or training sessions were considered important by 35.3% of respondents.

### 3.8 General feedback

Clinicians expressed a largely positive perception of wearable devices for the management of CCHD. The majority rated such devices as very promising (52.9%) or promising (41.2%), with only one neutral response (5.9%) and no skeptical responses.

When asked about design priorities for wearable devices, all respondents emphasized the importance of a user-friendly interface for patients and caregivers, underscoring the need for intuitive and accessible interaction. Other frequently cited features included real-time feedback and alerts (52.9%), integration with electronic health records (47.1%), and long battery life and durability (41.2%), indicating that clinicians value devices that balance clinical relevance, usability, and reliability in everyday use.

Regarding participation in device development, 41.2% of the clinicians expressed strong interest, 29.4% were conditionally willing, and 29.4% preferred not to participate.

Finally, the survey itself was considered valuable by clinicians, with 47.1% rating it as very important and 29.4% as moderately important. A smaller proportion of respondents considered it neutral (17.6%), while 5.9% rated it as moderately useless, and none viewed it as very useless.

## 4 Discussion

The present survey provides a structured overview of clinician perspectives on monitoring strategies in pediatric CCHD and offers actionable guidance for the development of a wearable multiparametric biosensor within the OrphaDev4Kids framework.

Current clinical monitoring was described as predominantly centered on imaging and basic physiological parameters, with limited availability of non-invasive approaches for biochemical assessment. In this context, clinicians identified additional parameters of interest, most notably metabolic markers such as lactate, which are currently accessible primarily through blood sampling. At the same time, non-invasive monitoring was reported to focus mainly on functional and physiological measures, such as activity levels and exercise tolerance. While existing monitoring approaches were generally perceived as effective, key limitations emerged, including poor integration with healthcare information systems and patient discomfort associated with device use.

Against this background, clinicians strongly prioritized the need for multiparametric monitoring, directly supporting the core concept of the OrphaDev4Kids project, which aims to develop a wearable biosensor capable of simultaneously capturing physiological and metabolic signals. Such an approach has the potential to move beyond episodic assessments and enable earlier identification of clinically relevant changes. Importantly, respondents emphasized that the value of remote monitoring extends beyond mortality reduction, encompassing morbidity reduction, detection of subtle deterioration, and prevention of acute decompensation events.

The burden of CCHD was also highlighted as extending well beyond clinical parameters, with substantial impact on daily life, including limitations in physical activity, frequent hospital visits, and psychological stress for patients and families. These findings further support the need for patient-friendly, home-based monitoring solutions, which may alleviate healthcare dependence and improve overall quality of life, as suggested by previous studies on remote physiologic monitoring in pediatric and chronic cardiac populations (12, 13).

In terms of target parameters for future devices, clinicians consistently identified oxygen saturation alongside metabolic markers such as lactate, as well as respiratory and hemodynamic variables. Notably, metabolic parameters remain largely absent from routine monitoring despite being recognized as clinically valuable. This gap represents a key unmet need and reinforces the rationale for developing wearable biosensors capable of continuous or semi-continuous metabolic assessment in real-world settings. In line with this, previous work has highlighted how continuous physiologic monitoring may enable earlier detection of intermittent or subtle deterioration that is unlikely to be captured through episodic assessments (1, 9). However, our findings indicate that continuous monitoring is not yet standardized in pediatric CCHD care, and currently available wearable technologies remain largely limited to heart rate–derived signals, underscoring the novelty of the proposed multiparametric approach.

At the same time, the survey revealed heterogeneous familiarity with wearable technologies, with limited awareness of devices capable of biochemical monitoring. This points to a clear knowledge and implementation gap in advanced biosensing within pediatric cardiology. While this may partly reflect variable exposure to emerging technologies, an aspect not directly assessed in this study, the coexistence of strong clinical interest and limited technological familiarity underscores the need for targeted education, effective dissemination strategies, and structured implementation pathways to enable successful clinical translation.

Beyond parameter selection, clinicians expressed clear and consistent expectations regarding device design, data management, and clinical integration. Wearability emerged as a central determinant of adoption, with comfort, unobtrusiveness, and ease of use identified as critical factors influencing patient adherence. These findings align with the concept of wearability described by Tandon et al. (9), which emphasizes the interplay between device functionality and usability in real-world settings. In pediatric populations, where long-term adherence is essential, integrating human-centered design principles is therefore a prerequisite for ensuring that technical performance translates into sustained clinical use.

Equally important, clinicians highlighted the need for efficient data handling and integration into clinical workflows. There was a clear preference for data outputs that prioritize clinically meaningful information, such as threshold-triggered alerts and concise summaries, rather than continuous raw data streams. This reflects the practical constraints of clinical environments and the risk of information overload. Consistent with prior literature, these findings underscore that wearable-derived data must be filtered, summarized, and integrated into electronic health record systems to be clinically actionable (9). The need for patient-specific thresholds further supports the development of customizable alerting strategies. In this context, artificial intelligence and machine learning may play a key role in transforming high-frequency, multiparametric data into actionable insights, although their application in this domain remains limited (9). Together, these results support a model in which wearable systems combine secure data integration, intelligent data reduction, and adaptive alerting to deliver clinically meaningful outputs while minimizing cognitive burden.

From an implementation perspective, the survey identified important organizational and infrastructural challenges. Integration of wearable data into routine care remains limited, with variability in institutional readiness and available resources for data review. These findings highlight that successful deployment of wearable technologies depends not only on device performance, but also on the existence of adequate frameworks for data governance, clinical oversight, and workflow integration. This is consistent with evolving European regulatory requirements for software as a medical device and real-world performance monitoring (MDCG 2019-11 rev.1, MDCG 2025-4 and MDR 2017/745) (14–16).

Data protection and privacy emerged as fundamental prerequisites for adoption. The level of concern expressed by clinicians underscores the importance of robust data governance frameworks, regulatory compliance, and privacy-by-design approaches. These considerations are particularly critical in pediatric populations, where ethical challenges related to consent and data protection are amplified (9, 17). As wearable devices increasingly collect and transmit sensitive, potentially identifiable health data, issues related to cybersecurity, data ownership, and responsible data use become central to their clinical deployment. Recent advances in secure cloud-based architectures for pediatric wearable monitoring demonstrate that technical solutions can effectively address these concerns through encrypted data transmission, automated data uploads that minimize manual handling errors, and GDPR-compliant storage infrastructure with granular access controls (18). Robust privacy frameworks and clear data exchange standards are therefore essential to ensure that data are managed securely and ethically, particularly in high-risk pediatric populations (19).

In addition to technical and organizational aspects, clinicians emphasized the importance of structured training and ongoing support. The preference for flexible, multimodal training formats reflects the realities of clinical practice and highlights that adoption of wearable technologies requires not only robust devices but also an enabling ecosystem of education, technical assistance, and continuous updates, as supported by previous literature (20).

Finally, the strong engagement with the survey itself reinforces the value of clinician involvement in early-stage device development. The clarity and consistency of the responses indicate that, although wearable devices are not yet fully embedded in routine practice, there is a well-defined set of clinical requirements regarding functionality, usability, and integration. This aligns with evidence showing that early stakeholder engagement improves the relevance and effectiveness of medical device development (21). Structured methods for gathering requirements, such as surveys and questionnaires, have been shown to effectively capture clinician needs and priorities, enabling development teams to align device features with real-world clinical workflows (22). This alignment between unmet clinical needs and desired device characteristics provides a robust foundation for further development. At the same time, the pattern of responses regarding direct involvement in device development suggests that early-stage engagement is feasible but should be designed with flexibility, acknowledging clinicians’ time constraints and the realities of clinical workload to ensure sustainable and meaningful participation.

While the survey highlights strong clinician enthusiasm for wearable devices, it is nevertheless important to emphasize that significant implementation barriers remain. Wearable biosensing in pediatric CCHD is currently limited by the absence of standardized pathways for continuous remote monitoring, challenges in integrating high-frequency physiological data into clinical workflows, and the risk of information overload without effective data filtering and prioritization strategies. A key translational bottleneck lies in the conversion of continuous multimodal signals into clinically actionable outputs, requiring robust data reduction, alert prioritization, and seamless integration into electronic health records within multidisciplinary care environments. These challenges are further compounded by heterogeneous institutional readiness for digital health adoption, including variability in data governance frameworks, post-market surveillance capacity, and operational responsibility for continuous data review. Taken together, these factors indicate that the clinical deployment of wearable biosensors in pediatric CCHD will depend not only on technological maturity, but critically on workflow redesign, regulatory alignment, and organizational infrastructure capable of supporting continuous data-driven care.

This study has some limitations. First, the relatively small sample size of survey participants may limit the generalizability of findings. However, it is important to consider that CCHD is a rare disease, and consequently, the number of clinicians with specialized expertise in this condition is inherently limited. Despite the small sample, the participants represented experienced specialists with in-depth knowledge of CCHD management, ensuring that the collected responses reflect clinical expertise rather than general opinions.

Second, the recruitment strategy, which relied on voluntary participation, may have introduced selection bias. Clinicians with greater interest in wearable devices or innovation may have been more likely to participate, potentially leading to an overestimation of technology acceptance and underrepresentation of skeptical or conservative perspectives. In addition, the sampling approach did not allow for control over participant characteristics, and the professional background of respondents was heterogeneous. While the majority were clinicians with expertise in pediatric cardiology, the inclusion of respondents at different career stages, including a medical student, may have introduced variability in the level of expertise and should be considered when interpreting the results.

Third, the geographical distribution of respondents was not systematically collected, and therefore cannot be precisely characterized. However, given that the OrphaDev4Kids Consortium is European in scope and that members of the Clinical Expert Committee involved in survey dissemination were predominantly based in European institutions, it is likely that the majority of respondents were European clinicians. As a result, the findings may primarily reflect European clinical practice and healthcare system organization, potentially limiting their applicability to other global contexts.

Finally, this study focused exclusively on clinicians’ perspectives and did not directly capture the views of patients with CCHD or their caregivers, who play a central role in disease management, particularly in home-based settings. To address this gap, additional surveys targeting patients and caregivers have been developed within the OrphaDev4Kids project and are currently being disseminated, with the aim of complementing the present findings and supporting a more comprehensive, user-centered approach to device development. Taken together, these limitations indicate that the results should be interpreted as exploratory and hypothesis-generating rather than definitive, and that further validation in larger, more diverse, and systematically characterized populations will be required.

Nevertheless, this study represents an important contribution to the emerging field of orphan medical device development, particularly in the context of CCHD. Future directions should focus on translating the clinical and technical requirements identified through this survey into iterative prototype refinement. In particular, the results support the development of a wearable, child-friendly biosensing device capable of continuous or semi-continuous monitoring of key parameters prioritized by clinicians, including oxygen saturation, heart rate, and metabolic markers, with lactate selected as a primary target based on survey responses and considerations of technical feasibility. This multiparametric approach directly reflects the needs expressed by respondents and aims to enable earlier detection of physiological and metabolic alterations preceding clinical deterioration. This process should be conducted in parallel with insights emerging from ongoing surveys targeting patients and caregivers, and should inform usability testing in real-world settings and early clinical feasibility studies, with particular attention to workflow integration, data summarization strategies, and long-term adherence. Subsequent development phases will need to address regulatory pathways for orphan medical devices, validation of clinical endpoints, and evaluation of impact on morbidity, healthcare utilization, and quality of life. Such a stepwise, clinician-informed approach will be essential to ensure that wearable biosensing devices can move beyond proof-of-concept and achieve sustainable clinical adoption in pediatric CCHD care.

## 5 Conclusion

This study provides the first structured overview of clinician expectations, needs, and perceived barriers regarding the use of wearable multiparametric biosensors for the management of pediatric patients with cyanotic congenital heart disease. The findings demonstrate strong clinical interest in non-invasive, multiparametric, and child-friendly wearable devices capable of supporting early detection of deterioration, particularly during infancy and other high-risk phases of care. At the same time, the results clearly show that successful adoption will depend not only on technological performance, but also on usability, patient compliance, data integration, training, and robust data governance.

Clinicians expressed a clear preference for actionable, summarized data supported by targeted alerts rather than continuous, unfiltered data streams. This highlights the need for wearable devices that are clinically meaningful, time-efficient, and seamlessly integrated into existing workflows. Comfort, intuitive design, and minimal disruption to daily life emerged as key determinants of long-term adherence in pediatric populations, reinforcing the importance of human-centered design in wearable devices.

Overall, these results confirm the presence of a well-defined unmet clinical need and provide concrete design requirements to guide the development of a wearable multiparametric biosensor for pediatric CCHD care. By integrating clinician-driven insights at this early stage, the OrphaDev4Kids project establishes a solid foundation for subsequent device refinement, clinical validation, and regulatory development, with the ultimate goal of enabling safer, more proactive, and patient-centered monitoring strategies for children living with complex congenital heart disease.

## Data Availability

The datasets presented in this study can be found in online repositories. The names of the repository/repositories and accession number(s) can be found below: https://doi.org/10.5281/zenodo.18623180 .
