# Supplementary material for: Clinical perspectives on wearable devices for pediatric cyanotic congenital heart disease: an expert survey to inform the early development of a multiparametric wearable biosensor
Source: Front Med (Lausanne). 2026 May 19;13:1819360. doi: 10.3389/fmed.2026.1819360 (PMC13226199; doi:10.3389/fmed.2026.1819360)
Supplement: Supplementary file 1 [file Table_1.docx]

# Survey on Cyanotic Congenital Heart Disease (CCHD)

This survey is part of Work Package 3 (WP3) under the OrphaDev4Kids European project. The project's primary goal is to create a robust innovation ecosystem in the field of orphan and paediatric medical devices (MDs), enhancing the value of research and development efforts for patients. This is achieved by supporting academic institutions, research centres, and SMEs focused on paediatric device development.

WP3 aims to design a wearable multiplexed biosensor for two specific purposes:

1. **Prevention of lactic acidosis** in children with unrepaired congenital heart defects.
2. **Monitoring and evaluating physiological responses** to pulmonary valve replacement in children with repaired congenital heart defects.

The survey seeks to collect valuable feedback to refine the development of the wearable multiplexed biosensor tailored for patients with **Cyanotic Congenital Heart Disease (CCHD)**. It is specifically designed to gather insights ensuring that the device meets technical, usability, and clinical effectiveness requirements.

Key details:

- **Anonymity:** data collection is entirely anonymous; no **personal data** and identifiers will be **processed**, therefore **the GDPR (Reg. UE 2016/679) is not applicable**.
- **Duration:** completing the survey takes approximately 20 minutes.

The **OrphaDev4Kids project** is coordinated by the Clinical Validation from Biopharmaceutical Findings (CVBF), in partnership with:

- Teddy European Network of Excellence for Paediatric Research (TEDDY)
- European Paediatric Translational Research Infrastructure (EPTRI)
- Gianni Benzi Foundation for Pharmacological Research (FGB)
- University of Bari
- IRCCS Eugenio Medea
- JEM TECH
- Children's Memorial Health Institute (IPCZD)
- Medical University Graz (MUG)

For further questions or information, please contact **Roberta Nossa** at [**roberta.nossa@lanostrafamiglia.it**](mailto:roberta.nossa@lanostrafamiglia.it).

Thank you for your contribution!

## Definitions

**Medical device:** in this survey, for medical device we mean any instrument, apparatus, appliance, software, implant, reagent, material or other article intended by the manufacturer to be used, alone or in combination, for human beings for one or more of the following specific medical purposes:

- diagnosis, prevention, monitoring, prediction, prognosis, treatment or alleviation of disease
- providing information by means of in vitro examination of specimens derived from the human body, including organ, blood and tissue donations.

**Biosensor:** is a device that can report the presence or activity of analytes using a biomolecular component providing specificity to the sensor by binding or interacting with the analyte and is able to cause a detectable change in mass, fluorescence, electric charge, or refractive index, and a transducer element, able to transform this interaction into a suitable electronic signal.

**Analyte:** a substance whose chemical constituents are being identified and measured.

**Single-use device:** means a device that is intended to be used on one individual during a single procedure.

## Expert profile and clinical context of CCHD management

1. Are you a caregiver for someone with Cyanotic Congenital Heart Disease (CCHD)? By "caregiver," we mean NON-PROFESSIONAL CAREGIVER, such as parents or guardians taking care of a child with CCHD, family members, friends or close relatives.

- Yes
- No

If the response was “Yes,” the platform automatically redirected participants to a separate survey designed for caregivers rather than clinicians. Responses from the caregiver survey were not included in the present analysis.

1. What is your professional role?

- Cardiologist
- Paediatrician
- Cardiac surgeon
- Nurse
- Physiotherapist
- Clinical psychologist
- Biochemist
- Biologist
- Biotechnologist
- Laboratory technician
- Engineer
- Other. If other, please specify: ________________

1. Have you ever dealt with Cyanotic Congenital Heart Diseases (CCHD) in your professional career?

- Yes
- No

1. Have you ever dealt with patients with Cyanotic Congenital Heart Diseases (CCHD) in your professional career?

- Yes
- No

If the response was “No” to both Question 3 and Question 4, the survey automatically skipped Questions 5, 6, and 8. Additionally, the survey displayed the sections “Validation and Testing” and “Data Analysis and Interpretation,” which were specifically designed for researchers and were therefore not included in the present analysis focused on clinicians. In this case, the section “Patient Compliance and Usability” was not displayed.

If the response was “Yes” to Question 3 and “No” to Question 4, the survey automatically skipped Questions 6 and 8. Additionally, the survey displayed the sections “Validation and Testing” and “Data Analysis and Interpretation,” which were specifically designed for researchers and were therefore not included in the present analysis focused on clinicians.

If the response was “Yes” to both Question 3 and Question 4, all questions and sections listed below were displayed in the survey.

1. How many years of experience do you have in working on CCHD?

- Less than 1 year
- 1 to 5 years
- 6 to 10 years
- More than 10 years

1. What is the age range of individuals with CCHD that you have encountered in your professional career? (Select all that apply)

- 0-1 year
- 1-5 years
- 6-12 years
- 13-17 years
- 18 years and older

1. Could you please share the types of CCHD cases you have worked on or studied in your professional experience, whether through clinical practice, research, or academic investigations?  (Select all that apply)

- Tetralogy of Fallot
- Transposition of the Great Arteries (TGA)
- Tricuspid Atresia
- Total Anomalous Pulmonary Venous Connection (TAPVC)
- Pulmonary Atresia
- Hypoplastic left heart syndrome (HLHS)
- Truncus arteriosus
- Ebstein’s Anomaly
- Double Outlet Right Ventricle (DORV)
- Single Ventricle Defects (e.g., Double Inlet Left Ventricle)
- Aortic Atresia
- Critical Pulmonary Stenosis
- Other. Please specify: ________________

1. Could you please estimate the number of patients you have encountered within each type of CCHD at your centre or across other centres?

**Tetralogy of Fallot**

- None
- 0-5
- 6-10
- 11-20
- 21-50
- 51-100
- More than 100

**Transposition of the Great Arteries (TGA)**

- None
- 0-5
- 6-10
- 11-20
- 21-50
- 51-100
- More than 100

**Tricuspid Atresia**

- None
- 0-5
- 6-10
- 11-20
- 21-50
- 51-100
- More than 100

**Total Anomalous Pulmonary Venous Return (TAPVC)**

- None
- 0-5
- 6-10
- 11-20
- 21-50
- 51-100
- More than 100

**Pulmonary Atresia**

- None
- 0-5
- 6-10
- 11-20
- 21-50
- 51-100
- More than 100

**Hypoplastic left heart syndrome** **(HLHS)**

- None
- 0-5
- 6-10
- 11-20
- 21-50
- 51-100
- More than 100

**Truncus arteriosus**

- None
- 0-5
- 6-10
- 11-20
- 21-50
- 51-100
- More than 100

**Ebstein’s Anomaly**

- None
- 0-5
- 6-10
- 11-20
- 21-50
- 51-100
- More than 100

**Double Outlet Right Ventricle (DORV)**

- None
- 0-5
- 6-10
- 11-20
- 21-50
- 51-100
- More than 100

**Single Ventricle Defects (e.g., Double Inlet Left Ventricle)**

- None
- 0-5
- 6-10
- 11-20
- 21-50
- 51-100
- More than 100

**Aortic Atresia**

- None
- 0-5
- 6-10
- 11-20
- 21-50
- 51-100
- More than 100

**Critical Pulmonary Stenosis**

- None
- 0-5
- 6-10
- 11-20
- 21-50
- 51-100
- More than 100

**Other (please specify)**: ________

- None
- 0-5
- 6-10
- 11-20
- 21-50
- 51-100
- More than 100

1. To your knowledge, at what stage of life or point in the treatment process could non-invasive or remote monitoring have the most significant impact on improving patient survival for each type of CCHD here mentioned?

**Tetralogy of Fallot**

- During infancy (before or after initial surgery)
- During childhood (ongoing treatment or post-surgical recovery)
- During adolescence (long-term management)
- During adulthood (lifelong follow-up)
- No impact at any stage
- Unsure

**Transposition of the Great Arteries**

- During infancy (before or after initial surgery)
- During childhood (ongoing treatment or post-surgical recovery)
- During adolescence (long-term management)
- During adulthood (lifelong follow-up)
- No impact at any stage
- Unsure

**Tricuspid Atresia**

- During infancy (before or after initial surgery)
- During childhood (ongoing treatment or post-surgical recovery)
- During adolescence (long-term management)
- During adulthood (lifelong follow-up)
- No impact at any stage
- Unsure

**Total Anomalous Pulmonary Venous Return**

- During infancy (before or after initial surgery)
- During childhood (ongoing treatment or post-surgical recovery)
- During adolescence (long-term management)
- During adulthood (lifelong follow-up)
- No impact at any stage
- Unsure

**Pulmonary Atresia**

- During infancy (before or after initial surgery)
- During childhood (ongoing treatment or post-surgical recovery)
- During adolescence (long-term management)
- During adulthood (lifelong follow-up)
- No impact at any stage
- Unsure

**Hypoplastic left heart syndrome**

- During infancy (before or after initial surgery)
- During childhood (ongoing treatment or post-surgical recovery)
- During adolescence (long-term management)
- During adulthood (lifelong follow-up)
- No impact at any stage
- Unsure

**Truncus arteriosus**

- During infancy (before or after initial surgery)
- During childhood (ongoing treatment or post-surgical recovery)
- During adolescence (long-term management)
- During adulthood (lifelong follow-up)
- No impact at any stage
- Unsure

**Ebstein’s Anomaly**

- During infancy (before or after initial surgery):
- During childhood (ongoing treatment or post-surgical recovery)
- During adolescence (long-term management)
- During adulthood (lifelong follow-up)
- No impact at any stage
- Unsure

**Double Outlet Right Ventricle (DORV)**

- During infancy (before or after initial surgery)
- During childhood (ongoing treatment or post-surgical recovery)
- During adolescence (long-term management)
- During adulthood (lifelong follow-up)
- No impact at any stage
- Unsure

**Single Ventricle Defects (e.g., Double Inlet Left Ventricle)**

- During infancy (before or after initial surgery)
- During childhood (ongoing treatment or post-surgical recovery)
- During adolescence (long-term management)
- During adulthood (lifelong follow-up)
- No impact at any stage
- Unsure

**Aortic Atresia**

- During infancy (before or after initial surgery)
- During childhood (ongoing treatment or post-surgical recovery)
- During adolescence (long-term management)
- During adulthood (lifelong follow-up)
- No impact at any stage
- Unsure

**Critical Pulmonary Stenosis**

- During infancy (before or after initial surgery)
- During childhood (ongoing treatment or post-surgical recovery)
- During adolescence (long-term management)
- During adulthood (lifelong follow-up)
- No impact at any stage
- Unsure

**Other (please specify)**: ________

- During infancy (before or after initial surgery)
- During childhood (ongoing treatment or post-surgical recovery)
- During adolescence (long-term management)
- During adulthood (lifelong follow-up)
- No impact at any stage
- Unsure

1. Which are the major challenges faced by patients with CCHD in their daily lives? (Select all that apply)

- Difficulty with physical activity
- Frequent medical visits
- Psychological stress
- Medication management
- Other. Please specify: ________________
- I am not able to answer

## Monitoring Methods

1. To your knowledge, which physiological/biochemical parameters are currently monitored in patients with CCHD as part of the standard care? (Select all that apply)

- Blood oxygen levels
- Heart rate and rhythm
- Blood pressure
- Hemodynamics (e.g., cardiac output)
- Arterial Blood Gases (ABG)
- Respiratory Rate
- Haemoglobin and Haematocrit
- Lactate Levels
- Pyruvic acid levels
- Biomarkers (e.g. Troponin, C-reactive protein, B-type Natriuretic Peptide, pro-B-type natriuretic peptide)
- Electrolytes (e.g., sodium, potassium)
- MRI/CT Scans
- Echocardiographic Findings
- Exercise Tolerance. Please specify with what it is monitored: ­­­­­­­­­­­­­­________________________
- Daily Activity Levels. Please specify with what they are monitored: ____________________
- Other. Please specify: ____________________
- I am not able to answer

1. In your opinion, which additional physiological/biochemical parameters, not currently monitored or not widely adopted, do you consider valuable for effectively managing and monitoring patients with CCHD? (Select all that apply)

- Blood oxygen levels
- Heart rate and rhythm
- Blood pressure
- Hemodynamics (e.g., cardiac output)
- Arterial Blood Gases (ABG)
- Respiratory Rate
- Haemoglobin and Haematocrit
- Lactate Levels
- Pyruvic acid levels
- Biomarkers (e.g. Troponin, C-reactive protein, B-type Natriuretic Peptide, pro-B-type natriuretic peptide)
- Electrolytes (e.g., sodium, potassium)
- MRI/CT Scans
- Echocardiographic Findings
- Exercise Tolerance
- Daily Activity Levels
- None
- Other. Please specify: ____________________
- I am not able to answer

1. To your knowledge, which biomarkers are currently monitored in patients with CCHD as part of the standard care? (Select all that apply)

- Troponin
- C-reactive protein (CRP)
- B-type Natriuretic Peptide (BNP)
- pro-B-type natriuretic peptide (pro BNP)
- Other. Please specify: _________________
- I am not able to answer

1. Which biomarkers do you think would be useful to monitor in CCHD patients, beyond the ones currently available through standard clinical methods? (Select all that apply)

- Troponin
- C-reactive protein (CRP)
- B-type Natriuretic Peptide (BNP)
- pro-B-type natriuretic peptide (pro BNP)
- None
- Other. Please specify: _________________
- I am not able to answer

1. Can the biomarker(s) identified to monitor the patient's condition be assessed through methods other than blood tests?

- Yes
- No
- I am not able to answer

1. If yes, which ones? (Select all that apply)

- Breath. Please specify the biomarker(s): ________________
- Saliva. Please specify the biomarker(s): ________________
- Urine. Please specify the biomarker(s): ________________
- Other (please specify): ________________

1. Among the physiological and biochemical parameters currently monitored in patients with CCHD as part of standard care, which ones are monitored using non-invasive methods? (Select all that apply)

- Blood oxygen levels
- Heart rate and rhythm
- Blood pressure
- Hemodynamics (e.g., cardiac output)
- Arterial Blood Gases (ABG)
- Respiratory Rate
- Haemoglobin and Haematocrit
- Lactate Levels
- Pyruvic acid levels
- Brain Natriuretic Peptide (BNP)/Pro-BNP
- Biomarkers (e.g. Troponin, C-reactive protein, B-type Natriuretic Peptide, pro-B-type natriuretic peptide)
- Electrolytes (e.g., sodium, potassium)
- MRI/CT Scans
- Echocardiographic Findings
- Exercise Tolerance
- Daily Activity Levels
- Other. Please specify: ___________________
- None
- I am not able to answer

1. How effective do you find current monitoring methods (both invasive or not) in providing accurate and timely information?

- Very effective
- Somewhat effective
- Neutral
- Somewhat ineffective
- Very ineffective
- I am not able to answer

1. What are the limitations of the current monitoring methods (both invasive or not) you use? (Select all that apply)

- Lack of accuracy
- Delayed feedback
- Limited range of monitored parameters
- Patient discomfort
- High cost
- Technical issues (e.g., connectivity problems)
- Lack of integration with other healthcare systems
- None
- Other. Please specify: __________
- I am not able to answer

1. To your knowledge, do you believe there are non-invasive monitoring methods, not yet widely adopted, that could serve as alternatives to the standard approaches for monitoring physiological/biochemical parameters in patients with CCHD?

- Yes. Please specify which parameter(s) and the corresponding non-invasive monitoring method(s): __________________
- No
- Unsure

1. Which advancements in wearable technology for monitoring heart conditions in CCHD would you prioritize for development? (Select all that apply)

- Miniaturized sensors
- Wireless connectivity
- Multiparametric monitoring
- Flexible and biocompatible materials
- Energy-efficient designs
- Advanced algorithms for early detection of anomalies and personalized insights based on patient-specific data
- Non-invasive biochemical monitoring
- Remote patient monitoring platforms
- Wearable ECG monitoring
- Paediatric-specific designs
- Other. Please specify: _____________________
- I am not able to answer

1. What types of data should the wearable biosensor/medical device prioritize collecting? (Select all that apply)

- Heart rate
- Blood oxygen saturation (SpO2)
- Blood pressure
- Activity levels and step counts
- Respiratory rate
- Sleep quality and duration
- Electrocardiogram (ECG) data
- Temperature trend
- Lactic acid or metabolic markers
- Other. Please specify: _____________________
- I am not able to answer

1. How often do you believe updates and alerts from the wearable biosensor or medical device should be delivered to optimize patient care and clinical decision-making?

- Real-time (as changes occur)
- Hourly
- Every few hours
- Daily summary
- Weekly summary
- Only when thresholds are crossed or abnormalities are detected
- Other. Please specify: _____________________

1. How important is access to historical data and trends over time for clinical decision-making?

- Extremely important: Trends over time are critical for making informed decisions
- Important: Historical data is helpful but not essential in every case
- Moderately important: Useful mainly for complex or long-term cases
- Not very important: Current data is usually sufficient
- Not important at all: Immediate clinical status is the sole focus

## Feedback on Wearable Technology

1. Considering the following wearable medical devices for monitoring heart conditions in paediatric cardiology, please indicate your level of familiarity with each

**Wearable ECG Monitors:**

- Very familiar
- Somewhat familiar
- Not familiar

**Wearable Blood Pressure Monitors:**

- Very familiar
- Somewhat familiar
- Not familiar

**Wearable Devices for Biochemical Monitoring (e.g., lactate, glucose, electrolytes):**

- Very familiar
- Somewhat familiar
- Not familiar

1. Please rate the importance of the following features for a wearable biosensor/medical device designed for patients with CCHD

**Real-time alerts for critical changes**

- Very important
- Somewhat important
- Not important
- I am not able to answer

**Remote monitoring capabilities**

- Very important
- Somewhat important
- Not important
- I am not able to answer

**User-friendly interface**

- Very important
- Somewhat important
- Not important
- I am not able to answer

**Data integration with healthcare providers**

- Very important
- Somewhat important
- Not important
- I am not able to answer

**Long battery life**

- Very important
- Somewhat important
- Not important
- I am not able to answer

**Comfortable and lightweight design**

- Very important
- Somewhat important
- Not important
- I am not able to answer

**Functionality (e.g. ease of use, usefulness, effectiveness, quality) in monitoring specific biomarkers (e.g., oxygen saturation, lactate levels)**

- Very important
- Somewhat important
- Not important
- I am not able to answer

**Additional functionalities. Please specify:** _______

- Very important
- Somewhat important
- Not important
- I am not able to answer

1. Do you think there are any other important features that a wearable biosensor/medical device designed for patients with CCHD should have?

- Yes. Please specify which ones and the importance (i.e. very important, somewhat important or not important): ______________
- No

## Patient Compliance and Usability

1. What factors influence patient compliance with wearable biosensor/medical devices?

(Please select up to 3 important options, if you can identify three. If you find only one or two options to be important, feel free to select just those)

- Comfort and design of the device
- Frequency of required use
- Accuracy and reliability of data
- Awareness of the health benefits provided by the device
- Parental involvement (for paediatric patients)
- Cost of the device and associated maintenance
- Technical support and ease of troubleshooting
- Psychological factors (e.g., stigma or self-image concerns)
- Other. Please specify: ________________

1. In your opinion, how important is the comfort and ease of use of a wearable medical device for its effective use in a clinical setting?

- Extremely important – it significantly impacts adherence and functionality
- Very important – comfort and usability are key factors for clinical efficiency
- Moderately important – some trade-offs are acceptable in clinical practice
- Not very important – clinical utility outweighs comfort considerations
- Not important at all – other factors are prioritized over comfort and ease of use

1. What challenges do you expect patients might face in using a wearable biosensor/medical device on a daily basis? (Please select up to 3 important options, if you can identify three. If you find only one or two options to be important, feel free to select just those)

- Discomfort during prolonged use
- Difficulty in operating or understanding the device
- Device interference with daily activities (e.g., work, exercise)
- Limited battery life or need for frequent charging
- Concerns about data privacy and security
- Lack of integration with other medical devices or platforms
- Cost of replacement parts or consumables
- Psychological resistance (e.g., feeling “monitored”)
- Social stigma related to wearing medical devices
- Other. Please specify: ________________

## Integration with Healthcare Systems

1. How do you currently integrate patient-generated health data into your clinical practice? (Select all that apply)

- Directly reviewed by healthcare providers during visits.
- Integrated into Electronic Health Records (EHRs) for review.
- Used mainly for research or long-term trend analysis.
- Reviewed by nursing or support staff before consultations.
- Not currently integrated due to technical or time constraints.
- Other. Please specify: __________________________

1. Would you find it useful for the biosensor data to be automatically shared with healthcare systems?

- Yes
- No

1. If so, how frequently?

- In real-time for continuous monitoring.
- With updates provided hourly.
- Only daily summaries are necessary.
- Only when thresholds or alerts are triggered.
- Other. Please specify: ______________

1. What information would you like to receive from the wearable biosensor/medical device to support your clinical practice? (Select all that apply)

- Alerts for critical conditions (e.g., low oxygen levels).
- Summaries of trends over a specified period (e.g., week, month).
- Data for specific time frames or activities (e.g., exercise).
- Integration of all available metrics into a single dashboard.
- Real-time patient feedback or notes about symptoms.
- Other. Please specify: ___________

1. Does your institution have facilities to handle the priority queries based on results from wearable medical devices?

- Yes, we have a dedicated team for reviewing wearable device data.
- Yes, but the system is not fully integrated with clinical workflows.
- No, our institution is too busy to manage real-time or frequent alerts.
- No, but we are considering developing such facilities.
- Other. Please specify: __________________

1. How much time would your clinical team realistically be willing to allocate to effectively use and integrate a wearable biosensor/medical device into patient care?

- Less than 5 minutes per patient
- 5-15 minutes per patient
- 15-30 minutes per patient
- 30 minutes to 1 hour per patient
- More than 1 hour per patient
- Not sure / Depends on the device and workflow integration

## Privacy and Security

1. How concerned are you about the privacy and security of the data collected by the wearable biosensor/medical device?

- Very concerned, strict security protocols are essential
- Somewhat concerned, provided standard protocols are followed
- Neutral, as long as the data benefits patient care
- Not very concerned, trust in existing systems
- Not concerned at all.

## Training and Support

1. What type of training and support would healthcare providers need to effectively use the wearable biosensor/medical device? (Select all that apply)

- Comprehensive in-person training
- Online video tutorials or e-learning modules
- Access to a technical support hotline or chat
- Regular updates and refresher courses
- No additional training is necessary
- Other. Please specify: _____________

1. How do you prefer to receive updates and support about new medical devices (e.g., online resources, in-person training, customer service)? (Select all that apply)

- Online resources (e.g., webinars, manuals)
- In-person workshops or training sessions
- On-demand customer support (e.g., hotline, email)
- Automatic updates integrated into the device or software
- Other. Please specify: ___________________________

## General Feedback

1. What is your overall impression of the potential of wearable technology in managing CCHD?

- Very promising, with great potential for improving outcomes
- Promising, but needs further development and validation
- Neutral, unsure about its impact
- Sceptical, there are significant challenges to overcome
- Not promising at all

1. Are there any other features or aspects you believe are crucial for the development of this wearable biosensor/medical device? (Select all that apply)

- User-friendly interface for patients and caregivers
- Integration with electronic health records
- Real-time feedback and alerts
- Long battery life and durability
- Other. Please specify: ______________

1. Would you be willing to participate in a trial or provide clinical insights during the development of this wearable biosensor/medical device?

- Yes, I am highly interested in participating. If you wish to contact us to contribute, you can write to [roberta.nossa@lanostrafamiglia.it](mailto:roberta.nossa@lanostrafamiglia.it) and [emilia.biffi@lanostrafamiglia.it](mailto:emilia.biffi@lanostrafamiglia.it). In this case, data processing will comply with applicable data protection provisions.
- Yes, but with some reservations or conditions. If you wish to contact us to contribute, you can write to [roberta.nossa@lanostrafamiglia.it](mailto:roberta.nossa@lanostrafamiglia.it) and [emilia.biffi@lanostrafamiglia.it](mailto:emilia.biffi@lanostrafamiglia.it). In this case, data processing will comply with applicable data protection provisions. Please specify your reservation/condition: ___________________
- No, I would prefer not to participate.

1. On a scale of 1 to 5, how would you rate the importance of this survey?

- 1: Very important.
- 2: Moderately important.
- 3: Neutral (neither important nor useless).
- 4: Moderately useless.
- 5: Very useless.
